# Temporal trends in incidence of time-loss injuries in four male professional North American sports over 13 seasons

**DOI:** 10.1038/s41598-021-87920-6

**Published:** 2021-04-15

**Authors:** Garrett S. Bullock, Elizabeth Murray, Jake Vaughan, Stefan Kluzek

**Affiliations:** 1 Centre for Sport, Exercise and Osteoarthritis Research Versus Arthritis, University of Oxford, Oxford, UK; 2 Nuffield Department of Orthopaedics, Rheumatology, and Musculoskeletal Sciences, University of Oxford, B4495, Oxford, OX3 7LD UK; 3 Department of Orthopaedic Surgery, Wake Forest School of Medicine, Winston-Salem, NC USA; 4 University of Nottingham, Nottingham, UK

**Keywords:** Health care, Health occupations, Medical research, Risk factors

## Abstract

Sports-related injuries increase healthcare cost burden, and in some instances have harmful long term physical and psychological implications. There is currently a lack of comprehensive data on temporal injury trends across professional North American sports. The purpose of this study was to compare temporal trends, according to incidence and time-loss injuries, by body part in professional baseball, basketball, football, and ice hockey. Public injury data from Major League Baseball, National Basketball Association, National Football League, and National Hockey League from 2007 to December 2019 were extracted and used. A mean of 62.49 injuries per 100 players per season was recorded for all professional sports. The groin/hip/thigh reported the greatest season proportional injury incidence for baseball, football, and ice hockey, with the groin/hip/thigh as the third highest injury incidence in basketball. When stratifying by more specific body part groupings, the knee demonstrated the greatest injury proportional incidence for basketball, football, and ice hockey, with the knee as the third highest proportional injury incidence for baseball. There was an increased in basketball ankle injuries following 2011–2012 season. Football and ice hockey reported the greatest concussion proportion incidence, with football demonstrating an increase in concussions over time, and a substantial increase in concussions from the 2014 to 2015 season. These publicly extracted data and findings can be used as a shared resource for professional baseball, basketball, football, and ice hockey for future individual and across sport collaborations concerning resource allocation and decision making in order to improve player health.

## Introduction

It is estimated that annually more than 4000 athletes play in the top professional leagues for baseball, basketball, football, and ice hockey (Major League Baseball (MLB), National Basketball Association (NBA), National Football League, (NFL) and National Hockey League (NHL))^1^. Professional sport requires high physiologic^2^, biomechanical^3^, and psychological stress^4^, which can increase and predispose a professional athlete to greater injury risk^3,4^. Injuries in professional sports have been reported as high as 3.61 per 1000 athlete exposures in baseball, 19.3 in basketball, 64.7 in football, and 49.4 in ice hockey^5–8^. Sports-related injuries have a healthcare cost^9^, and in some instances have long term physical^10^ and psychological implications^11^. For years now, sports medicine professionals and organizations have attempted to implement injury risk reduction strategies and promote safer participation^12–14^. A four step sequence for injury prevention by van Mechelen et al. includes Ref.^15^: incidence and severity of sports injuries, the recognition of risk factors and injury mechanisms, identification of potential modulators and evaluation of preventive measures. The time trend analysis of injury patterns is an essential step in this process for both risk factors identification and evaluating the effect of potential modulators.

Many interventions have been shown to reduce specific injury risk^12^. For example, incorporating Nordic eccentric hamstring training has been shown to reduce hamstring strains in professional baseball players^12^. Further, implementing proprioceptive and balance training reduced ankle sprains and low back pain in professional basketball players^16^. However, while injury risk reduction strategies are effective^12,16^, these risk reduction strategies may be adopted in one sport, but another sport with similar injuries may not adopt the same injury mitigation interventions, potentially creating disparate injury rates^17^.

There is currently a lack of comprehensive data on temporal injury trends (vary across seasons) across the four major professional North American sports mentioned above. This is despite the wide use of professional sports reporting of not only players characteristics, sports specific performance, sports transactions but also major injuries in addition to coverage of the events themselves. With relevant publicly available player performance and injury data, professional sport presents a unique public platform to identify potential injury and illness risk factors in well-conditioned and healthy individuals.

Deciphering time-loss injury temporal trends across these four major professional sports can be used to compare and contrast injuries between the sports. These open access data and findings can be used by any person interested in investigating injuries in these professional sports, as a shared resource for each individual sport, but also as a beginning to collaborate on improving shared injury trends between sports. The purpose of this study was to compare temporal trends, according to incidence and time-loss injuries, by body part in professional baseball, basketball, football, and ice hockey.

## Material and methods

### Study design

We estimated and evaluated temporal trends of incidence in time-loss injuries using datasets from the Major League Baseball, National Basketball Association, National Football League, and National Hockey League as maintained by the website (https://www.prosportstransactions.com). Temporal trend was defined as incidence in time-loss injuries over the course of multiple seasons and stratified by sport and body part. Following consultation with the University Institutional Review Board, institution ethical approval was not needed for this study due to the public nature of the data.

### Injury and illness definitions

An injury was defined as tissue damage or other derangement of normal physical function reported by a player and his team that occurred during any team sponsored activity or event and was followed by at least one game missed^18,19^. An illness was defined as a complaint or disorder reported by a player and his team, not related to injury, and resulted in at least one game missed^18,19^. Examples of illness include physical, mental, or wellbeing^19^.

### Injury and illness inclusion and exclusion criterion

Season timeframe was based on the date of the first official game or competition, and ended with the last playoff game or competition. Off season and preseason injuries were not considered for analyses due to the sparseness and inconsistency in the data, and the inability to corroborate if injuries were sustained during team sponsored activities. Injuries were based on body area categories defined by the Orchard Sports Injury Classification System, which is further described in the next section^20,21^.

### Injury classification

Wherever possible, we defined body areas anatomically as either joints or segments. However, we made exceptions based on common clinical presentations. For example, concussion was an example which does not fit to a specific joint or segment, but represents a specific body part subcategory. This is the same approach recently published by the International Olympic Committee Injury and Illness Epidemiology Consensus Group^19^. We were not able to code more than one injury from a singular injury event, as time-loss injury was coded for the most severe injury. In order to try to account for this issue, sub analyses utilizing sport injury/illness diagnostic classification and coding were performed^19^.

Using the Orchard Sports Injury Classification System, injured body parts were initially head/neck, shoulder/arm/elbow, forearm/wrist/hand, trunk/back/buttock, groin/hip/thigh, knee, lower leg/Achilles tendon, ankle, foot/toe, and concussion. Following initial body part classification, body parts were further grouped^20,21^.

### Data extraction

Data were downloaded using a reproducible process, often referred to as ‘data scraping,’ in which a programming language extracts data from web sites, into a human-readable output^22^. Data extraction was conducted in R version 3.5.1, using the rvest, tm, and xml2 packages. Each sport’s data was extracted from the Pro Sports transactions web page, with only missed games due to injury tab selected. Please refer to Appendix 1 for a list of the sports websites. Sport data was extracted from 1980 to December 1, 2019. Please refer to Appendix 2 for complete code.

### Data reduction

Injury data was checked for consistency through visual inspection and grouping data by year. Data was observed to be inconsistent prior to 2007. Inconsistencies included missed days (missing specific game days), months, or teams. As a result, data prior to 2007 was dismissed from the data set.

Injury data from the beginning of each professional season in 2007 (MLB: April 1, 2007; NBA: October 30, 2007; NFL: September 6, 2007; NHL: September 9, 2007) to December 1, 2019 was utilized for this study. The beginning date of each MLB, NBA, NFL, and NHL season was confirmed through official data reports. The end of the season was coded as one month following the final playoff game. It was determined through consensus that including injury reports one month following the season would capture season inflicted injuries. For full dataset, please refer to Appendix 3.

### External validation

This extracted dataset is based on systematic collection of published, publicly available injury reports over time. However, the blanket assertions on the good validity of those records cannot be made without testing. For that reason, two independent examiners (EM and JV) used a random number generator to pick 100 players from each injury sport dataset and looked for externally published confirmation of those reported injuries. Each examiner externally queried each player, date, and injury type through internet search and then compared these findings to the injury data set. 90% of randomly selected records were confirmed in other reports, with some variation between sports: baseball is 83%, basketball 80%, football and ice hockey 98% each (Appendix 4). It was concluded that the data set demonstrated good reliability in reporting injuries within the four sports.

### Statistical analyses

Injury count data were converted to seasonal injury incidence proportion. Proportional injury incidence was calculated by number of injuries divided by number of total players for each individual season. Injury incidence was then multiplied by a 100 to include each individual seasonal injury incidence per 100 players. 95% confidence intervals were reported for injury incidence proportion^23^. Further, mean difference, minimum, and maximum proportional injury incidence per 100 players were also calculated to compare to the individual seasonal analyses. Following main analyses, sensitivity analyses were performed to calculate proportional injury incidence over aggregated five-season intervals to evaluate the influence of seasonal outliers. The number of players per season were queried through baseball-reference.com, basketball-reference.com, pro-football-reference.com, and hockey-reference.com. For full list of number of players per season, please refer to Appendix 5. Data were descriptively analysed through means, counts, and visual plotting. All analyses were performed in R version 3.5.1 (R Core Team (2020). R: A language and environment for statistical computing. R Foundation for Statistical Computing, Vienna, Austria. URL https://www.R-project.org/), with mean, count, and mean difference analyses performed in the package Hmisc and all plots created through the package ggplot2.

### Ethics approval and consent to participate

Due to the publicly available nature of the data, institution ethics approval was not needed for this study.

## Results

A total of 54,944 individual injuries were recorded: 17,065 for baseball, 13,930 for basketball, 10,019 for football, and 13,930 for ice hockey. Over thirteen seasons, 51,861 players participated in these professional sports. The average number of overall injuries for all four sports was 62.49 injuries per 100 players per season. Table 1 reports the injury incidence proportion by body part by sport. Table 2 reports the injury incidence proportion by further body part groupings by sport. For injury incidence proportion in a tabular format by year and sport and body part, please refer to Appendices 5–8. Further detail of injury incidence proportion by year, sport, and body, please refer to injury incidence comparison by sport and season section.

**Table 1:** Mean proportional incidence with 95% confidence intervals per 100 players body part and sport.

Body part	Baseball			Basketball			Football			Ice Hockey		
	Incidence proportion	95% CI		Incidence proportion	95% CI		Incidence proportion	95% CI		Incidence proportion	95% CI	
Ankle	1.79	1.76	1.81	13.81	13.68	13.94	4.44	4.40	4.49	1.80	1.77	1.83
Concussion	0.37	0.37	0.38	1.68	1.64	1.72	3.45	3.42	3.49	3.17	3.13	3.21
Foot/toe	1.97	1.95	2.00	6.27	6.18	6.35	2.13	2.11	2.16	2.40	2.36	2.44
Forearm/wrist/hand	7.46	7.41	7.52	5.69	5.61	5.77	1.17	1.15	1.18	3.71	3.66	3.75
Groin/hip/thigh	8.65	8.59	8.71	12.10	11.98	12.22	6.51	6.46	6.56	7.44	7.37	7.51
Head/neck	2.88	2.85	2.92	2.21	2.17	2.26	1.11	1.09	1.12	4.68	4.63	4.74
Knee	3.45	3.42	3.49	13.94	13.81	14.07	5.95	5.90	6.00	4.08	4.03	4.13
Lower leg/achilles tendon	1.73	1.71	1.76	4.21	4.15	4.28	1.34	1.32	1.36	0.16	0.15	0.18
Arm/shoulder/elbow	6.92	6.86	6.97	4.94	4.86	5.01	2.28	2.25	2.31	3.42	3.38	3.47
Trunk/back/buttock	6.78	6.73	6.83	8.01	7.92	8.11	2.38	2.35	2.41	7.94	7.87	8.02

*95% CI* 95% confidence interval.

**Table 2:** Mean proportional incidence with 95% confidence intervals per 100 players by body part and sport.

Injury	Baseball			Basketball			Football			Ice hockey		
	Incidence proportion	95% CI		Incidence proportion	95% CI		Incidence proportion	95% CI		Incidence proportion	95% CI	
Abdominal	0.37	0.36	0.38	0.61	0.60	0.62	0.20	0.19	0.21	0.32	0.31	0.33
Abductor	0.08	0.07	0.09	0.43	0.42	0.44	0.18	0.16	0.19	0.00	0.00	0.00
Achilles	0.24	0.23	0.25	1.47	1.43	1.50	1.39	1.37	1.41	0.11	0.09	0.13
Adductor	1.77	1.75	1.80	2.60	2.55	2.65	0.00	0.00	0.00	3.13	3.09	3.17
Ankle	1.79	1.76	1.81	13.81	13.68	13.94	4.44	4.40	4.49	1.80	1.77	1.83
Arm	0.40	0.40	0.41	0.25	0.23	0.26	0.19	0.18	0.20	0.52	0.52	0.53
Back	4.70	4.65	4.74	6.77	6.68	6.86	0.93	0.92	0.94	2.05	2.01	2.08
Bicep	0.31	0.30	0.32	0.21	0.19	0.23	0.10	0.09	0.11	0.11	0.10	0.13
Calf	1.16	1.15	1.18	2.26	2.21	2.31	0.98	0.97	1.00	0.19	0.17	0.21
Chest	0.21	0.20	0.22	0.33	0.32	0.35	0.39	0.38	0.39	0.22	0.20	0.23
Collarbone	0.08	0.06	0.09	0.20	0.18	0.22	0.10	0.09	0.12	0.14	0.13	0.16
Concussion	0.37	0.37	0.38	1.68	1.64	1.72	3.45	3.42	3.49	3.17	3.13	3.21
Elbow	2.59	2.56	2.62	1.18	1.15	1.21	0.35	0.34	0.36	0.23	0.22	0.24
Eye	0.27	0.26	0.28	0.53	0.52	0.53	0.12	0.10	0.13	0.48	0.48	0.49
Face	0.17	0.15	0.18	0.27	0.26	0.29	0.07	0.06	0.08	0.83	0.81	0.84
Fibula	0.08	0.06	0.09	0.28	0.26	0.29	0.15	0.14	0.17	0.13	0.12	0.15
Finger	1.49	1.47	1.51	1.05	1.03	1.08	0.18	0.17	0.19	0.67	0.66	0.68
Foot	1.28	1.26	1.30	4.11	4.05	4.18	1.59	1.57	1.62	2.14	2.11	2.18
Forearm	0.93	0.91	0.94	0.25	0.23	0.27	0.18	0.17	0.20	0.16	0.14	0.17
Gluteus	0.10	0.09	0.11	0.19	0.17	0.21	0.07	0.06	0.08	0.12	0.10	0.14
Hamstring	3.84	3.80	3.88	3.18	3.12	3.23	3.71	3.67	3.75	0.30	0.29	0.31
Hand	1.78	1.75	1.80	1.30	1.27	1.33	0.46	0.46	0.47	1.71	1.68	1.74
Head	0.88	0.86	0.89	0.76	0.74	0.78	0.53	0.53	0.54	2.00	1.97	2.04
Heel	0.29	0.28	0.30	0.63	0.61	0.64	0.13	0.12	0.14	0.12	0.10	0.13
Hernia	0.17	0.15	0.18	0.30	0.28	0.31	0.15	0.13	0.16	0.20	0.19	0.22
Hip	1.08	1.07	1.10	2.87	2.81	2.92	0.42	0.42	0.43	1.16	1.14	1.18
Intercostal	0.10	0.09	0.11	0.00	0.00	0.00	0.00	0.00	0.00	0.30	0.29	0.32
Jaw	0.13	0.11	0.14	0.39	0.37	0.40	0.13	0.12	0.14	0.00	0.00	0.00
Knee	3.45	3.42	3.49	13.37	13.25	13.50	5.95	5.90	6.00	3.78	3.73	3.83
Leg	0.66	0.65	0.66	0.96	0.94	0.98	0.22	0.21	0.23	2.03	2.00	2.07
Neck	1.54	1.52	1.56	0.68	0.67	0.70	0.66	0.66	0.67	0.85	0.84	0.87
Oblique	1.31	1.29	1.33	0.28	0.26	0.29	0.11	0.09	0.12	0.24	0.23	0.25
Patella	0.08	0.07	0.09	0.74	0.72	0.76	0.56	0.55	0.56	0.11	0.09	0.13
Quadricep	1.09	1.07	1.11	1.49	1.46	1.52	0.00	0.00	0.00	0.16	0.14	0.17
Rib	0.63	0.62	0.64	0.66	0.64	0.67	0.38	0.37	0.39	0.47	0.46	0.47
Shin	0.33	0.33	0.34	0.38	0.36	0.39	0.09	0.08	0.10	0.12	0.10	0.13
Shoulder	3.42	3.39	3.46	3.50	3.44	3.56	1.75	1.72	1.77	2.50	2.46	2.54
Thigh	0.12	0.11	0.13	0.78	0.77	0.80	0.37	0.36	0.38	0.26	0.25	0.27
Thumb	1.23	1.21	1.25	1.34	1.31	1.38	0.24	0.23	0.25	0.20	0.18	0.21
Toe	0.43	0.43	0.44	1.51	1.47	1.54	0.50	0.50	0.50	0.17	0.15	0.19
Tricep	0.27	0.26	0.28	0.35	0.33	0.36	0.10	0.09	0.12	0.11	0.09	0.13
Wrist	2.05	2.02	2.07	1.90	1.86	1.94	0.18	0.17	0.19	0.81	0.80	0.83

*95% CI* 95% confidence interval.

### Baseball

The highest baseball injury incidence was to the back, with a mean proportional injury incidence from the 2007 through 2019 season was 4.69 per 100 players. Hamstring injuries had the second highest mean proportional injury incidence of 3.84, followed by a 3.45 mean knee proportional injury incidence, 3.43 mean shoulder proportional injury incidence, and a 2.59 mean elbow proportional injury incidence per 100 players (Table 1). The highest peak proportional injury incidence was to the back in the 2014 season, with a peak mean proportional injury incidence of 6.06 per 100 players (Appendix 5). Please refer to Appendix 5 for mean proportional injury incidence by body part for the 2007 through 2019 seasons.

### Basketball

The highest basketball proportional injury incidence was to the ankle, with a mean proportional injury incidence from 2007–2008 through the 2019–2020 seasons was 13.82 per 100 players. A drop in proportional injury incidence was observed at the beginning of the 2019–2020 season prior to end of data collection on December 1, 2019. Knee injuries had the second highest mean proportional injury incidence of 13.37 per 100 players, followed by a 6.77 mean back proportional injury incidence, 4.11 mean foot proportional injury incidence, a 3.18 mean hamstring proportional injury incidence, and a 2.60 mean adductor proportional injury incidence per 100 players (Table 1). There was an NBA lockout during the 2011 season; which resulted in the season not starting until January 2012. The highest peak basketball mean proportional injury incidence was to the knee in the 2013–2014 season, with a peak mean proportional injury incidence of 17.43 per 100 players (Appendix 6). Please refer to Appendix 6 for mean injury incidence by body part for the 2007–2008 through 2019–2020 seasons.

### Football

The highest football proportional injury incidence was to the knee, with mean proportional injury incidence from 2007–2008 through the 2019–2020 seasons was found to be 5.95 per 100 players. Ankle injuries had the second highest mean proportional injury incidence of 4.44 per 100 players, followed by a 3.45 mean concussion proportional injury incidence, 1.75 mean shoulder proportional injury incidence, and a 1.59 mean foot injury proportional incidence per 100 players (Table 1). The highest peak football mean proportional injury incidence was to the knee in the 2015–2016 season, with a peak mean proportional injury incidence of 7.07 per 100 players (Appendix 7). Please refer to Appendix 7 for mean proportional injury incidence by body part for the 2007–2008 through 2019–2020 seasons.

### Ice hockey

The highest ice hockey proportional injury incidence was to the knee, with a mean proportional injury incidence from 2007–2008 through the 2019–2020 seasons was found to be 3.78 per 100 players. Concussion had the second highest mean proportional injury incidence of 3.17 per 100 players, followed by a mean adductor proportional injury incidence of 3.13, and a 1.80 mean ankle proportional injury incidence per 100 players (Table 1). There was an NHL lockout in the beginning of the 2012–2013 season, which results in the season not starting until January 2013. The highest peak ice hockey mean proportional injury incidence was to the knee, with a peak mean proportional injury incidence of 10.33 per 100 players in the 2007–2008 season (Appendix 8). Please refer to Appendix 8 for mean proportional injury incidence by body part for the 2007–2008 through 2019–2020 seasons.

### Injury incidence comparison by sport and season

**Figure 1:**
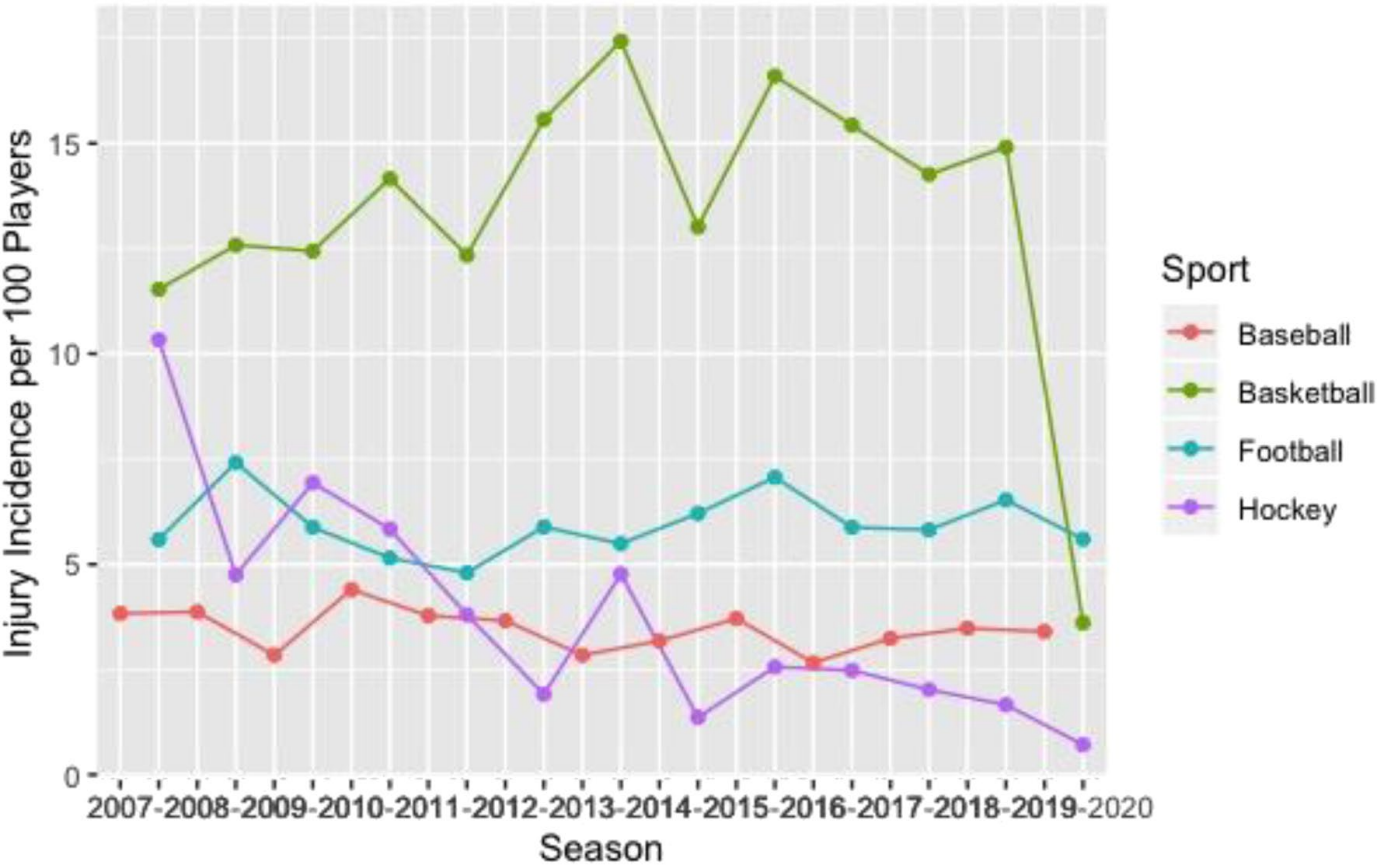
Knee time-loss injury by sport.

**Figure 2:**
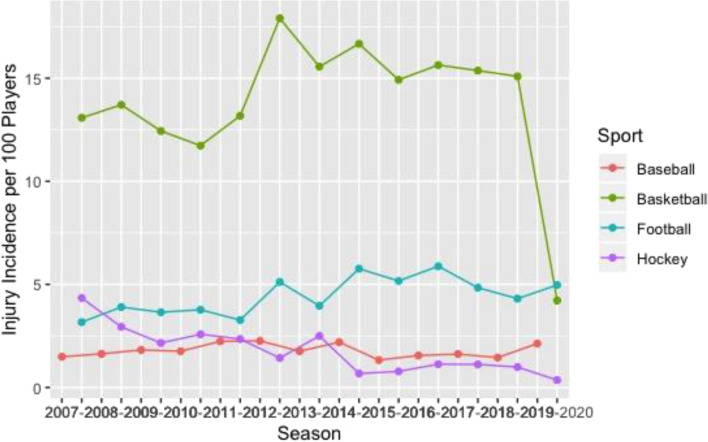
Ankle time-loss injury by sport.

**Figure 3:**
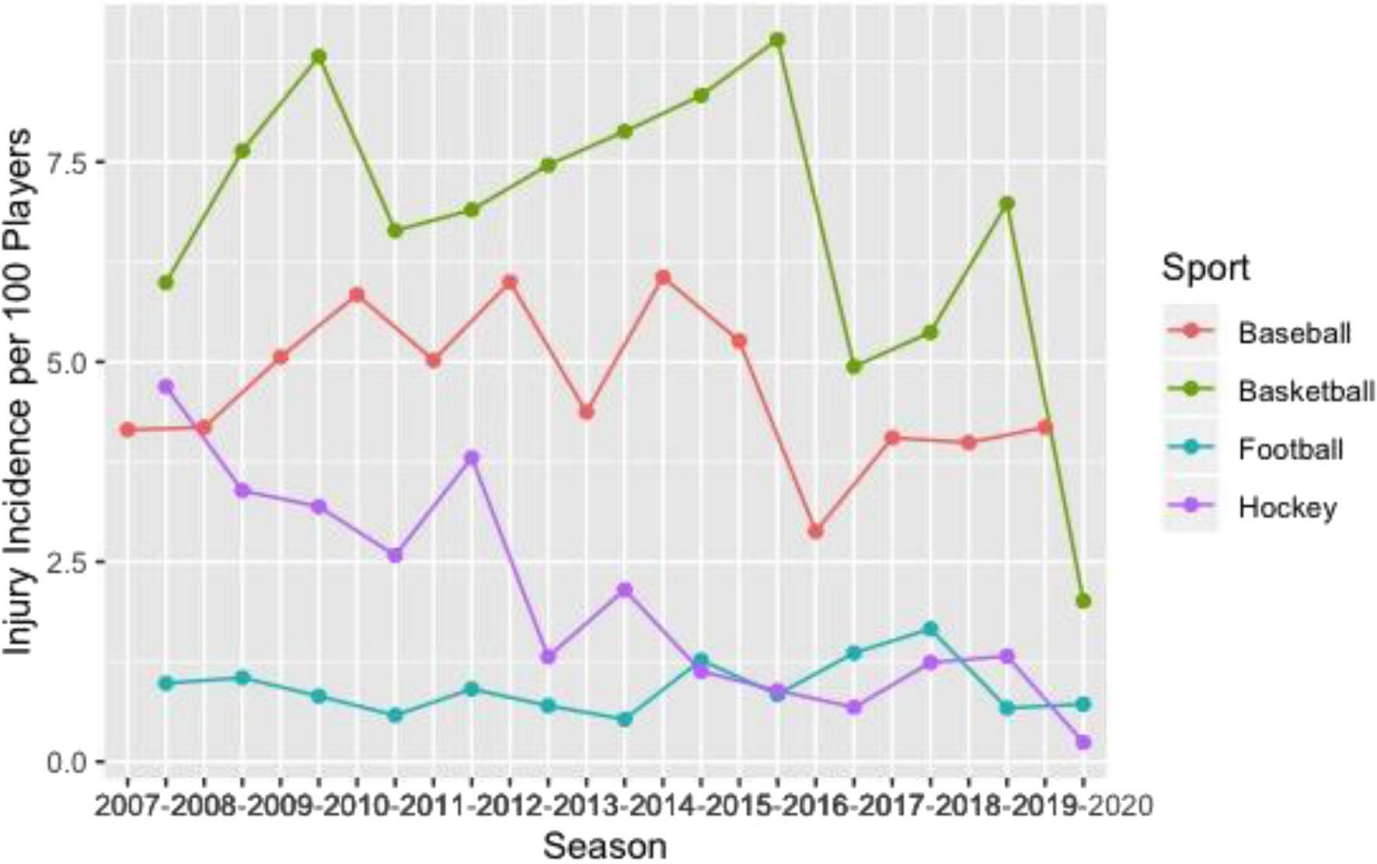
Back time-loss injury by sport.

**Figure 4:**
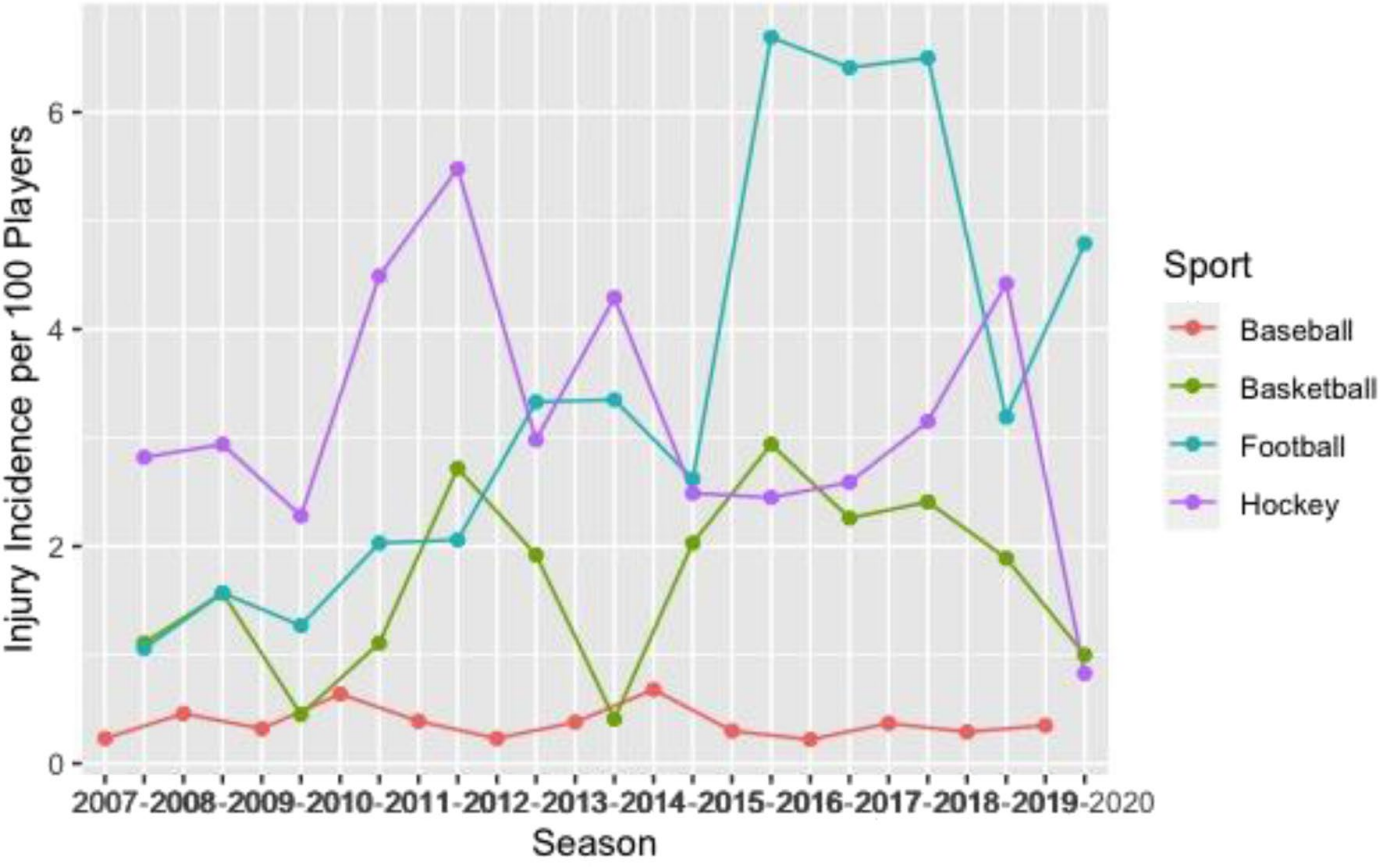
Concussion time-loss injury by sport.

**Figure 5:**
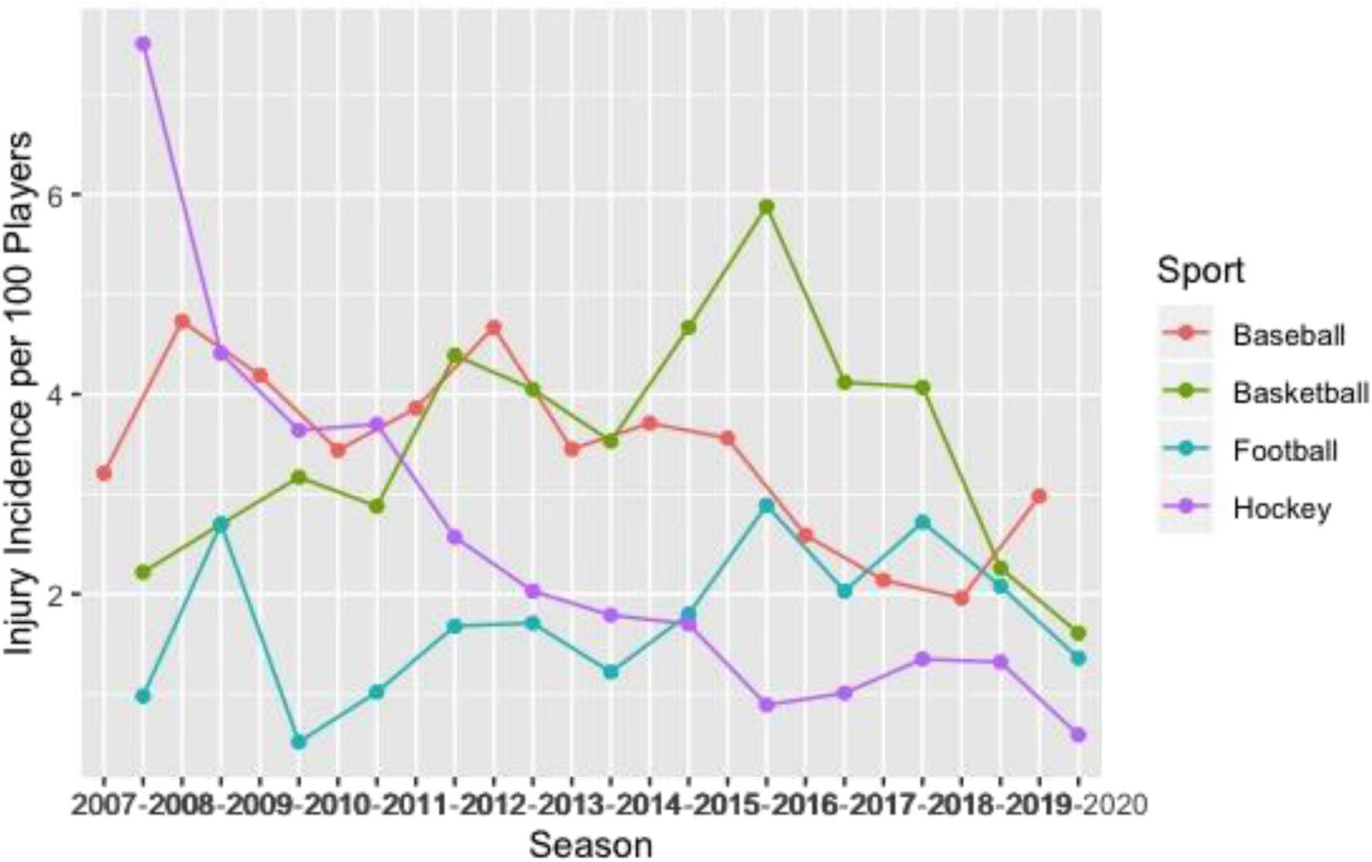
Shoulder time-loss injury by sport.

Basketball had the greatest knee (Fig. 1), ankle (Fig. 2), and back (Fig. 3) time-loss injury incidence compared to baseball, football, and ice hockey. Football and ice hockey had the greatest concussion incidence (Fig. 4). Football’s mean concussion incidence increased from 2.62 to 6.69 per 100 players between the 2014–2015 and 2015–2016 seasons. There was a marked decrease in mean shoulder time-loss injury incidence in ice hockey, demonstrated by a mean decrease of 6.19 per 100 players over the 12-year reporting period (Fig. 5).

### Sensitivity analyses

Basketball reported the greatest mean consecutive five-season proportional injury incidence for the knee (17.43) Appendix Figure 9), ankle (17.91) (Appendix Figure 10), and back (9.03) (Appendix Figure 11) compared to baseball, football, and ice hockey. Football and ice hockey had the greatest concussion proportional incidence per 100 players (Football: 4.82; Ice Hockey: 3.61) (Appendix Figure 12). Baseball, basketball, football, and ice hockey five-season proportional injury incidence by body part is depicted in Appendices 13, 14, 15, and 16, respectively. The mean difference was similar between season and five-season analyses across body parts (Appendices 17 and 18). For example, ankle proportional injury incidence per 100 players mean difference comparing season and 5-year analyses were: Baseball (Season: 0.38, Five Year: 0.38), Basketball (Season: 3.41, Five Year: 2.93), Football (Season: 1.07, Five Year: 1.08), and Ice Hockey (Season: 1.29, Five Year: 1.29).

## Discussion

### Summary

The highest proportional injury incidence was for the groin/hip/thigh in baseball, football, and ice hockey, with the groin/hip/thigh as the third highest injury incidence in basketball. When stratifying by more specific body part groupings, the knee demonstrated the greatest injury proportional incidence for basketball, football, and ice hockey, with the knee as the third highest proportional injury incidence for baseball. Football and ice hockey reported the greatest concussion proportion incidence, with football demonstrating an increase in concussions over time, and a substantial increase in concussions from the 2014 to 2015 season. Similar results were found using consecutive five-season increments.

### Groin/hip/thigh injuries

When stratifying injuries by body parts in Table 1, the groin/hip/thigh reported the greatest injury incidence for baseball, football, and ice hockey, and the third highest for basketball. The thigh was the second highest injury incidence for football and ice hockey in former studies^7,8^. In contrast, previous literature has reported the greatest injury incidence to the shoulder and elbow in baseball, but when stratified by position players and pitchers, hamstring injuries demonstrated the second highest injury incidence in position players^5^. The greatest injury incidence to a specific body part to the groin/hip/thigh for baseball, basketball, football, and ice hockey has been to the hamstring^5–8^. In a systematic review, no individual risk factors were identified for hamstring injuries^24^. Further research is required to understand if there are different injury mechanisms for groin/hip/thigh injuries between baseball, basketball, football, and ice hockey.

### Knee injuries

When grouping by more specific body parts in Table 2, knee injuries demonstrated the greatest proportional injury incidence for basketball, football, and ice hockey. Baseball knee injuries were reported for as the third highest injured body part, which supports previous epidemiologic literature^5,25,26^. Basketball reported the highest proportional seasonal injury incidence, with 8 more knee injuries per 100 players, compared to the second highest proportional knee injury incidence in football. Professional basketball knee injuries have reported greater injury severity in comparison to other injures^27^. Further, almost 50% of asymptomatic professional basketball players have knee lesions on MRI imaging^28^. Within our data there was a marked spike in knee injuries between 2012–2013 and 2013–2014 seasons. In 2012, the NBA instituted an anti-flopping rule, which prohibited players from bailing on charges and blocks, which may have contributed to this spike in injuries^29^. Previous literature has observed that the most common knee injury mechanism of injury in basketball and baseball is noncontact^30,31^, compared to contact injury in football and ice hockey^8,32^. While knee injuries demonstrated the greatest incidence for all sports but baseball, the mechanisms of injury may differ, potentially altering the implications of these injuries between sports.

### Concussion

Football and ice hockey reported the greatest seasonal proportional concussion incidence, which is supported by previous literature^33,34^. Professional sports have instituted concussion teams, return to play protocols, and rules and regulations to improve player safety^34,35^. Our NFL concussion data demonstrated a steady increase in concussion incidence since 2009, with the NFL concussion incidence increasing by 31.6% in the 2015 seasons compared to the 2014 season. Since 2009, the NFL has instituted league wide concussion protocols^36^. Due to this increase in incidence, the NFL instituted harsher penalties and fines to teams for not following concussion protocols^37^. However, within our data, there was not a marked decrease in concussion incidence for the 2016 season. Our NBA concussion data demonstrated an increase in concussions beginning in the 2011 season, except for a decreased in the 2013 season. This supports the initiation of the NBA concussion protocols beginning in the 2011–2012 season^35^. It should be noted that concussion incidence did not change throughout the reporting period. Baseball concussion incidence has been minimal in previous literature^5,38^, despite a concussion protocol instituted in the 2011–2012 season^39^.

### Sensitivity analysis

Analysing injury incidence over five consecutive seasons instead of per season, demonstrated similar findings. Five-year season variability and overall mean proportional injury incidence were similar between analyses. Previous literature has reported similar stability in findings for multiple professional sports^5,34,35^. However, within our data, there were specific injury variances, such as within basketball knee injury and football concussion, that were captured in both analyses. These data suggest that implementing either 5 year or season to season variances in injuries may still capture the overall trends in injury data.

### Strengths and limitations

Generalisability of these findings is limited to the four professional men’s North American leagues and cannot be assumed to be reproduceable in semi-professional, amateur, female, or youth sport. This study assessed temporal trends over a 12-year span, increasing the validity of these findings. This study utilized publicly available data, and analysed these data through open access tools, supporting the transparency of the methods and results. Study methodology included external validation of a random sample of the data with other publicly available data, increasing the strength of these results. Due to using publicly available data, the ability to identify missing data is not possible and therefore the completeness of recording can be questioned. However, these data report time-loss injuries and it is unlikely that severe injuries are missed. This work does not report on injuries which did not lead to missed training or competition, decreasing the overall completeness of these injury data. There is also a potential bias for under-reporting more severe potential time-loss injuries if occurring at the same time as another injury. Athlete exposure could not be quantified. As different athletes may have different exposure to sport, these data cannot calculate injury incidence nor injury rates, decreasing the clinical utility of these data. At this stage the data did not allow for time-loss severity to be analysed, which decreases the clinical interpretability of these findings. In some instances, more specific injury reporting was not possible (e.g., shoulder injury instead of rotator cuff injury), which also decreases the clinical interpretability of these findings.

### Future research

These findings instigate future research. The open data extracted for this study did not include exposure or severity information. Future research is needed to improve the open data extraction methods, access, and validation methods. All four sports reported a high injury incidence at multiple body parts. Future investigations are necessary to understand the long-term implications of these injuries on athlete physical and mental health and the prevention of these injuries. Finally, many body part injury incidence proportions were similar between baseball, basketball, football, and ice hockey. Research is needed to investigate if shared resource allocation and decision making between these four professional men’s sports leagues can improve overall player health.

## Conclusion

When stratifying by body parts, the groin/hip/thigh demonstrated the greatest proportional injury incidence for baseball, football, and ice hockey. When stratifying by more specific body parts, basketball, football, and ice hockey reported the greatest proportional injury incidence to the knee, while baseball reported the greatest proportional injury incidence to the back. Football and ice hockey reported the greatest concussion proportion incidence. These open data and results can be used as a shared resource for professional baseball, basketball, football, and ice hockey leagues for future collaborations and investigation concerning player injury and health.

## Supplementary information

Supplementary Information 1.

Supplementary Information 2.

## Data Availability

All data relevant to the study are included are uploaded as supplementary information. Please refer to Appendix 2 for the data extraction code and Appendix 3 for original data.

## Code availability

Please refer to Appendix 2 for data extraction code.
