# Supplementary material for: Temporal trends in incidence of time-loss injuries in four male professional North American sports over 13 seasons
Source: Sci Rep. 2021 Apr 15;11:8278. doi: 10.1038/s41598-021-87920-6 (PMC8050171; doi:10.1038/s41598-021-87920-6)
Supplement: Supplementary file 2 — Supplementary Information 2. [file 41598_2021_87920_MOESM2_ESM.docx]

**Temporal Trends in Incidence of Time-loss Injuries in Four Male Professional North American Sports Over 13 Seasons**

# Garret S. Bullock, PT, DPT,^1,2,3^ Elizabeth Murray,^4^ Jake Vaughan ,^4^ Stefan Kluzek, MRCP, M.Sc, D.Phil^1,2,4^

1. Centre for Sport, Exercise and Osteoarthritis Research *Versus Arthritis,* University of Oxford, United Kingdom
2. Nuffield Department of Orthopaedics, Rheumatology, and Musculoskeletal Sciences, University of Oxford, Oxford, United Kingdom
3. Department of Orthopaedic Surgery, Wake Forest School of Medicine, Winston-Salem, North Carolina, USA
4. University of Nottingham, Nottingham, UK.

Appendix 1. List of Websites for Data Scraping

Baseball:

<https://www.prosportstransactions.com/baseball/index.htm>

Basketball:

<https://www.prosportstransactions.com/basketball/index.htm>

Football:

<https://www.prosportstransactions.com/football/index.htm>

Hockey:

<https://www.prosportstransactions.com/hockey/index.htm>

Appendix 2. Data Extraction R Code

#Data Scraping

library(robotstxt)

library(xml2)

library(rvest)

library(stringr)

library(dplyr)

library(tidyr)

library(purrr)

#Table iterations

#/html/body/div[4]/table[1]

#/html/body/div[4]/table[1]

#/html/body/div[4]/table[1]

#Same Table HTML code for every table

#Web page

#https://www.prosportstransactions.com/baseball/Search/SearchResults.php?Player=&Team=&BeginDate=&EndDate=&InjuriesChkBx=yes&submit=Search&start=525

#https://www.prosportstransactions.com/baseball/Search/SearchResults.php?Player=&Team=&BeginDate=&EndDate=&InjuriesChkBx=yes&submit=Search&start=550

#https://www.prosportstransactions.com/baseball/Search/SearchResults.php?Player=&Team=&BeginDate=&EndDate=&InjuriesChkBx=yes&submit=Search&start=575

#https://www.prosportstransactions.com/baseball/Search/SearchResults.php?Player=&Team=&BeginDate=&EndDate=&InjuriesChkBx=yes&submit=Search&start=600

#Web pages go up by 25

#Multiple pages:

url_base <- "https://www.prosportstransactions.com/baseball/Search/SearchResults.php?Player=&Team=&BeginDate=&EndDate=&InjuriesChkBx=yes&submit=Search&start=%d"

map_df(seq(0,100, 25), function(i) {

print(sprintf(url_base, i))

data.frame(html_nodes(read_html(sprintf(url_base, i)), xpath = '/html/body/div[4]/table[1]') %>%

html_table(header = T))

}) -> testing

#works

testing <- testing %>%

mutate(

Acquired = substring(Acquired, 3),

Relinquished = substring(Relinquished, 3)

)

#Baseball

url_base <- "https://www.prosportstransactions.com/baseball/Search/SearchResults.php?Player=&Team=&BeginDate=&EndDate=&InjuriesChkBx=yes&submit=Search&start=%d"

map_df(seq(0,19475, 25), function(i) {

print(i)

data.frame(html_nodes(read_html(sprintf(url_base, i)), xpath = '/html/body/div[4]/table[1]') %>%

html_table(header = T))

}) -> baseball_injury

baseball_injury <- baseball_injury %>%

mutate(

Acquired = substring(Acquired, 3),

Relinquished = substring(Relinquished, 3)

)

write.csv(baseball_injury, "/Users/student/Desktop/baseball_injury.csv")

#Basketball

url_basket <- "https://www.prosportstransactions.com/basketball/Search/SearchResults.php?Player=&Team=&BeginDate=&EndDate=&InjuriesChkBx=yes&Submit=Search&start=%d"

map_df(seq(0,27900, 25), function(i) {

print(i)

data.frame(html_nodes(read_html(sprintf(url_basket, i)), xpath = '/html/body/div[4]/table[1]') %>%

html_table(header = T))

}) -> basketball_injury

basketball_injury <- basketball_injury %>%

mutate(

Acquired = substring(Acquired, 3),

Relinquished = substring(Relinquished, 3)

)

write.csv(basketball_injury, "/Users/student/Desktop/basketball_injury.csv")

#Football

url_football <- "https://www.prosportstransactions.com/football/Search/SearchResults.php?Player=&Team=&BeginDate=&EndDate=&InjuriesChkBx=yes&submit=Search&start=%d"

map_df(seq(0,12175, 25), function(i) {

print(i)

data.frame(html_nodes(read_html(sprintf(url_football, i)), xpath = '/html/body/div[4]/table[1]') %>%

html_table(header = T))

}) -> football_injury

football_injury <- football_injury %>%

mutate(

Acquired = substring(Acquired, 3),

Relinquished = substring(Relinquished, 3)

)

write.csv(football_injury, "/Users/student/Desktop/football_injury.csv")

#Hockey

url_hockey <- "https://www.prosportstransactions.com/hockey/Search/SearchResults.php?Player=&Team=&BeginDate=&EndDate=&InjuriesChkBx=yes&submit=Search&start=%d"

map_df(seq(0,21850, 25), function(i) {

print(i)

data.frame(html_nodes(read_html(sprintf(url_hockey, i)), xpath = '/html/body/div[4]/table[1]') %>%

html_table(header = T))

}) -> hockey_injury

hockey_injury <- hockey_injury %>%

mutate(

Acquired = substring(Acquired, 3),

Relinquished = substring(Relinquished, 3)

)

write.csv(hockey_injury, "/Users/student/Desktop/hockey_injury.csv")

Appendix 3. Extracted Data Set

Please see CSV file for the extracted raw data.

Appendix 4. Number of Players Per Season

Baseball:

| Season | Number of Players |
| --- | --- |
| 2005 | 1237 |
| 2006 | 1242 |
| 2007 | 1278 |
| 2008 | 1291 |
| 2009 | 1266 |
| 2010 | 1249 |
| 2011 | 1295 |
| 2012 | 1284 |
| 2013 | 1304 |
| 2014 | 1320 |
| 2015 | 1349 |
| 2016 | 1353 |
| 2017 | 1358 |
| 2018 | 1379 |
| 2019 | 1410 |

Basketball:

| Season | Number of Players |
| --- | --- |
| 2004-2005 | 464 |
| 2005-2006 | 458 |
| 2006-2007 | 458 |
| 2007-2008 | 451 |
| 2008-2009 | 445 |
| 2009-2010 | 442 |
| 2010-2011 | 452 |
| 2011-2012 | 478 |
| 2012-2013 | 469 |
| 2013-2014 | 482 |
| 2014-2015 | 492 |
| 2015-2016 | 476 |
| 2016-2017 | 486 |
| 2017-2018 | 540 |
| 2018-2019 | 530 |
| 2019-2020 | 498 |

Football:

| Season | Number of players |
| --- | --- |
| 2004-2005 | 1327 |
| 2005-2006 | 1320 |
| 2006-2007 | 1298 |
| 2007-2008 | 1324 |
| 2008-2009 | 1334 |
| 2009-2010 | 1343 |
| 2010-2011 | 1379 |
| 2011-2012 | 1313 |
| 2012-2013 | 1290 |
| 2013-2014 | 1312 |
| 2014-2015 | 1337 |
| 2015-2016 | 1315 |
| 2016-2017 | 1327 |
| 2017-2018 | 1323 |
| 2018-2019 | 1347 |
| 2019-2020 | 1107 |

Hockey:

| Season | Number of players |
| --- | --- |
| 2005-2006 | 870 |
| 2006-2007 | 858 |
| 2007-2008 | 852 |
| 2008-2009 | 885 |
| 2009-2010 | 879 |
| 2010-2011 | 891 |
| 2011-2012 | 894 |
| 2012-2013 | 839 |
| 2013-2014 | 886 |
| 2014-2015 | 882 |
| 2015-2016 | 898 |
| 2016-2017 | 888 |
| 2017-2018 | 890 |
| 2018-2019 | 906 |
| 2019-2020 | 845 |

Appendix 5. Baseball Injury Incidence by Body Part per Season

| Season | Injured Body Part | Count | Seasonal Injury Incidence per 100 Players |
| --- | --- | --- | --- |
| 2007 | Abdominal | 5 | 0.39 |
| 2007 | Achilles | 1 | 0.08 |
| 2007 | Adductor | 22 | 1.72 |
| 2007 | Ankle | 19 | 1.49 |
| 2007 | Arm | 6 | 0.47 |
| 2007 | Back | 53 | 4.15 |
| 2007 | Bicep | 3 | 0.23 |
| 2007 | Calf | 12 | 0.94 |
| 2007 | Chest | 3 | 0.23 |
| 2007 | Concussion | 3 | 0.23 |
| 2007 | Elbow | 34 | 2.66 |
| 2007 | Eye | 3 | 0.23 |
| 2007 | Finger | 16 | 1.25 |
| 2007 | Foot | 12 | 0.94 |
| 2007 | Forearm | 17 | 1.33 |
| 2007 | Hamstring | 49 | 3.83 |
| 2007 | Hand | 12 | 0.94 |
| 2007 | Head | 5 | 0.39 |
| 2007 | Heel | 4 | 0.31 |
| 2007 | Hernia | 6 | 0.47 |
| 2007 | Hip | 8 | 0.63 |
| 2007 | Knee | 49 | 3.83 |
| 2007 | Leg | 6 | 0.47 |
| 2007 | Neck | 14 | 1.1 |
| 2007 | Oblique | 20 | 1.56 |
| 2007 | Patella | 1 | 0.08 |
| 2007 | Quadricep | 11 | 0.86 |
| 2007 | Rib | 10 | 0.78 |
| 2007 | Shin | 3 | 0.23 |
| 2007 | Shoulder | 41 | 3.21 |
| 2007 | Thigh | 1 | 0.08 |
| 2007 | Thumb | 18 | 1.41 |
| 2007 | Toe | 3 | 0.23 |
| 2007 | Tricep | 4 | 0.31 |
| 2007 | Wrist | 20 | 1.56 |
| 2008 | Abdominal | 3 | 0.23 |
| 2008 | Abductor | 1 | 0.08 |
| 2008 | Achilles | 1 | 0.08 |
| 2008 | Adductor | 23 | 1.78 |
| 2008 | Ankle | 21 | 1.63 |
| 2008 | Arm | 3 | 0.23 |
| 2008 | Back | 54 | 4.18 |
| 2008 | Bicep | 3 | 0.23 |
| 2008 | Calf | 13 | 1.01 |
| 2008 | Chest | 3 | 0.23 |
| 2008 | Concussion | 6 | 0.46 |
| 2008 | Elbow | 26 | 2.01 |
| 2008 | Eye | 8 | 0.62 |
| 2008 | Face | 1 | 0.08 |
| 2008 | Finger | 27 | 2.09 |
| 2008 | Foot | 9 | 0.7 |
| 2008 | Forearm | 11 | 0.85 |
| 2008 | Gluteus | 1 | 0.08 |
| 2008 | Hamstring | 49 | 3.8 |
| 2008 | Hand | 20 | 1.55 |
| 2008 | Head | 4 | 0.31 |
| 2008 | Hernia | 1 | 0.08 |
| 2008 | Hip | 16 | 1.24 |
| 2008 | Knee | 50 | 3.87 |
| 2008 | Leg | 9 | 0.7 |
| 2008 | Neck | 13 | 1.01 |
| 2008 | Oblique | 8 | 0.62 |
| 2008 | Quadricep | 20 | 1.55 |
| 2008 | Rib | 8 | 0.62 |
| 2008 | Shin | 1 | 0.08 |
| 2008 | Shoulder | 61 | 4.73 |
| 2008 | Thigh | 3 | 0.23 |
| 2008 | Thumb | 15 | 1.16 |
| 2008 | Toe | 2 | 0.15 |
| 2008 | Tricep | 4 | 0.31 |
| 2008 | Wrist | 16 | 1.24 |
| 2009 | Abdominal | 3 | 0.24 |
| 2009 | Achilles | 1 | 0.08 |
| 2009 | Adductor | 30 | 2.37 |
| 2009 | Ankle | 23 | 1.82 |
| 2009 | Arm | 4 | 0.32 |
| 2009 | Back | 64 | 5.06 |
| 2009 | Bicep | 4 | 0.32 |
| 2009 | Calf | 18 | 1.42 |
| 2009 | Chest | 3 | 0.24 |
| 2009 | Collarbone | 1 | 0.08 |
| 2009 | Concussion | 4 | 0.32 |
| 2009 | Elbow | 44 | 3.48 |
| 2009 | Eye | 5 | 0.39 |
| 2009 | Face | 1 | 0.08 |
| 2009 | Finger | 20 | 1.58 |
| 2009 | Foot | 10 | 0.79 |
| 2009 | Forearm | 10 | 0.79 |
| 2009 | Gluteus | 2 | 0.16 |
| 2009 | Hamstring | 58 | 4.58 |
| 2009 | Hand | 18 | 1.42 |
| 2009 | Head | 10 | 0.79 |
| 2009 | Heel | 5 | 0.39 |
| 2009 | Hernia | 1 | 0.08 |
| 2009 | Hip | 17 | 1.34 |
| 2009 | Knee | 36 | 2.84 |
| 2009 | Leg | 11 | 0.87 |
| 2009 | Neck | 21 | 1.66 |
| 2009 | Oblique | 15 | 1.18 |
| 2009 | Quadricep | 8 | 0.63 |
| 2009 | Rib | 6 | 0.47 |
| 2009 | Shin | 5 | 0.39 |
| 2009 | Shoulder | 53 | 4.19 |
| 2009 | Thumb | 13 | 1.03 |
| 2009 | Toe | 6 | 0.47 |
| 2009 | Tricep | 4 | 0.32 |
| 2009 | Wrist | 26 | 2.05 |
| 2010 | Abdominal | 8 | 0.64 |
| 2010 | Achilles | 4 | 0.32 |
| 2010 | Adductor | 17 | 1.36 |
| 2010 | Ankle | 22 | 1.76 |
| 2010 | Arm | 8 | 0.64 |
| 2010 | Back | 73 | 5.84 |
| 2010 | Bicep | 4 | 0.32 |
| 2010 | Calf | 17 | 1.36 |
| 2010 | Chest | 2 | 0.16 |
| 2010 | Concussion | 8 | 0.64 |
| 2010 | Elbow | 43 | 3.44 |
| 2010 | Eye | 4 | 0.32 |
| 2010 | Face | 1 | 0.08 |
| 2010 | Finger | 22 | 1.76 |
| 2010 | Foot | 19 | 1.52 |
| 2010 | Forearm | 15 | 1.2 |
| 2010 | Gluteus | 1 | 0.08 |
| 2010 | Hamstring | 44 | 3.52 |
| 2010 | Hand | 17 | 1.36 |
| 2010 | Head | 15 | 1.2 |
| 2010 | Heel | 6 | 0.48 |
| 2010 | Hip | 12 | 0.96 |
| 2010 | Intercostal | 1 | 0.08 |
| 2010 | Jaw | 1 | 0.08 |
| 2010 | Knee | 55 | 4.4 |
| 2010 | Leg | 9 | 0.72 |
| 2010 | Neck | 23 | 1.84 |
| 2010 | Oblique | 22 | 1.76 |
| 2010 | Quadricep | 19 | 1.52 |
| 2010 | Rib | 14 | 1.12 |
| 2010 | Shin | 4 | 0.32 |
| 2010 | Shoulder | 43 | 3.44 |
| 2010 | Thigh | 1 | 0.08 |
| 2010 | Thumb | 14 | 1.12 |
| 2010 | Toe | 12 | 0.96 |
| 2010 | Tricep | 1 | 0.08 |
| 2010 | Wrist | 28 | 2.24 |
| 2011 | Abdominal | 8 | 0.62 |
| 2011 | Achilles | 5 | 0.39 |
| 2011 | Adductor | 28 | 2.16 |
| 2011 | Ankle | 29 | 2.24 |
| 2011 | Arm | 7 | 0.54 |
| 2011 | Back | 65 | 5.02 |
| 2011 | Bicep | 5 | 0.39 |
| 2011 | Calf | 20 | 1.54 |
| 2011 | Chest | 1 | 0.08 |
| 2011 | Collarbone | 1 | 0.08 |
| 2011 | Concussion | 5 | 0.39 |
| 2011 | Elbow | 39 | 3.01 |
| 2011 | Eye | 5 | 0.39 |
| 2011 | Face | 5 | 0.39 |
| 2011 | Finger | 25 | 1.93 |
| 2011 | Foot | 23 | 1.78 |
| 2011 | Forearm | 13 | 1 |
| 2011 | Gluteus | 1 | 0.08 |
| 2011 | Hamstring | 45 | 3.47 |
| 2011 | Hand | 27 | 2.08 |
| 2011 | Head | 17 | 1.31 |
| 2011 | Heel | 5 | 0.39 |
| 2011 | Hernia | 3 | 0.23 |
| 2011 | Hip | 18 | 1.39 |
| 2011 | Intercostal | 1 | 0.08 |
| 2011 | Jaw | 1 | 0.08 |
| 2011 | Knee | 49 | 3.78 |
| 2011 | Leg | 6 | 0.46 |
| 2011 | Neck | 24 | 1.85 |
| 2011 | Oblique | 23 | 1.78 |
| 2011 | Quadricep | 20 | 1.54 |
| 2011 | Rib | 11 | 0.85 |
| 2011 | Shin | 10 | 0.77 |
| 2011 | Shoulder | 50 | 3.86 |
| 2011 | Thigh | 5 | 0.39 |
| 2011 | Thumb | 20 | 1.54 |
| 2011 | Toe | 8 | 0.62 |
| 2011 | Tricep | 5 | 0.39 |
| 2011 | Wrist | 35 | 2.7 |
| 2012 | Abdominal | 7 | 0.55 |
| 2012 | Achilles | 6 | 0.47 |
| 2012 | Adductor | 31 | 2.41 |
| 2012 | Ankle | 29 | 2.26 |
| 2012 | Arm | 7 | 0.55 |
| 2012 | Back | 77 | 6 |
| 2012 | Bicep | 7 | 0.55 |
| 2012 | Calf | 14 | 1.09 |
| 2012 | Chest | 6 | 0.47 |
| 2012 | Collarbone | 1 | 0.08 |
| 2012 | Concussion | 3 | 0.23 |
| 2012 | Elbow | 49 | 3.82 |
| 2012 | Eye | 5 | 0.39 |
| 2012 | Face | 5 | 0.39 |
| 2012 | Fibula | 1 | 0.08 |
| 2012 | Finger | 20 | 1.56 |
| 2012 | Foot | 17 | 1.32 |
| 2012 | Forearm | 10 | 0.78 |
| 2012 | Hamstring | 70 | 5.45 |
| 2012 | Hand | 28 | 2.18 |
| 2012 | Head | 15 | 1.17 |
| 2012 | Heel | 4 | 0.31 |
| 2012 | Hip | 10 | 0.78 |
| 2012 | Intercostal | 2 | 0.16 |
| 2012 | Jaw | 1 | 0.08 |
| 2012 | Knee | 47 | 3.66 |
| 2012 | Leg | 11 | 0.86 |
| 2012 | Neck | 23 | 1.79 |
| 2012 | Oblique | 26 | 2.02 |
| 2012 | Quadricep | 16 | 1.25 |
| 2012 | Rib | 8 | 0.62 |
| 2012 | Shin | 7 | 0.55 |
| 2012 | Shoulder | 60 | 4.67 |
| 2012 | Thigh | 1 | 0.08 |
| 2012 | Thumb | 15 | 1.17 |
| 2012 | Toe | 8 | 0.62 |
| 2012 | Tricep | 3 | 0.23 |
| 2012 | Wrist | 31 | 2.41 |
| 2013 | Abdominal | 4 | 0.31 |
| 2013 | Abductor | 1 | 0.08 |
| 2013 | Achilles | 6 | 0.46 |
| 2013 | Adductor | 26 | 1.99 |
| 2013 | Ankle | 23 | 1.76 |
| 2013 | Arm | 4 | 0.31 |
| 2013 | Back | 57 | 4.37 |
| 2013 | Bicep | 8 | 0.61 |
| 2013 | Calf | 18 | 1.38 |
| 2013 | Collarbone | 1 | 0.08 |
| 2013 | Concussion | 5 | 0.38 |
| 2013 | Elbow | 39 | 2.99 |
| 2013 | Eye | 5 | 0.38 |
| 2013 | Fibula | 1 | 0.08 |
| 2013 | Finger | 22 | 1.69 |
| 2013 | Foot | 18 | 1.38 |
| 2013 | Forearm | 12 | 0.92 |
| 2013 | Hamstring | 52 | 3.99 |
| 2013 | Hand | 27 | 2.07 |
| 2013 | Head | 11 | 0.84 |
| 2013 | Heel | 2 | 0.15 |
| 2013 | Hernia | 1 | 0.08 |
| 2013 | Hip | 12 | 0.92 |
| 2013 | Intercostal | 1 | 0.08 |
| 2013 | Knee | 37 | 2.84 |
| 2013 | Leg | 8 | 0.61 |
| 2013 | Neck | 15 | 1.15 |
| 2013 | Oblique | 18 | 1.38 |
| 2013 | Quadricep | 16 | 1.23 |
| 2013 | Rib | 10 | 0.77 |
| 2013 | Shin | 6 | 0.46 |
| 2013 | Shoulder | 45 | 3.45 |
| 2013 | Thigh | 1 | 0.08 |
| 2013 | Thumb | 23 | 1.76 |
| 2013 | Toe | 6 | 0.46 |
| 2013 | Tricep | 7 | 0.54 |
| 2013 | Wrist | 33 | 2.53 |
| 2014 | Abdominal | 7 | 0.53 |
| 2014 | Adductor | 19 | 1.44 |
| 2014 | Ankle | 29 | 2.2 |
| 2014 | Arm | 4 | 0.3 |
| 2014 | Back | 80 | 6.06 |
| 2014 | Bicep | 2 | 0.15 |
| 2014 | Calf | 20 | 1.52 |
| 2014 | Chest | 1 | 0.08 |
| 2014 | Concussion | 9 | 0.68 |
| 2014 | Elbow | 46 | 3.48 |
| 2014 | Eye | 2 | 0.15 |
| 2014 | Face | 3 | 0.23 |
| 2014 | Finger | 17 | 1.29 |
| 2014 | Foot | 19 | 1.44 |
| 2014 | Forearm | 12 | 0.91 |
| 2014 | Hamstring | 51 | 3.86 |
| 2014 | Hand | 32 | 2.42 |
| 2014 | Head | 14 | 1.06 |
| 2014 | Heel | 1 | 0.08 |
| 2014 | Hernia | 2 | 0.15 |
| 2014 | Hip | 14 | 1.06 |
| 2014 | Intercostal | 1 | 0.08 |
| 2014 | Jaw | 3 | 0.23 |
| 2014 | Knee | 42 | 3.18 |
| 2014 | Leg | 8 | 0.61 |
| 2014 | Neck | 21 | 1.59 |
| 2014 | Oblique | 21 | 1.59 |
| 2014 | Quadricep | 19 | 1.44 |
| 2014 | Rib | 7 | 0.53 |
| 2014 | Shin | 6 | 0.45 |
| 2014 | Shoulder | 49 | 3.71 |
| 2014 | Thigh | 1 | 0.08 |
| 2014 | Thumb | 12 | 0.91 |
| 2014 | Toe | 4 | 0.3 |
| 2014 | Tricep | 3 | 0.23 |
| 2014 | Wrist | 25 | 1.89 |
| 2015 | Abdominal | 3 | 0.22 |
| 2015 | Achilles | 5 | 0.37 |
| 2015 | Adductor | 30 | 2.22 |
| 2015 | Ankle | 18 | 1.33 |
| 2015 | Arm | 7 | 0.52 |
| 2015 | Back | 71 | 5.26 |
| 2015 | Bicep | 2 | 0.15 |
| 2015 | Calf | 11 | 0.82 |
| 2015 | Chest | 2 | 0.15 |
| 2015 | Concussion | 4 | 0.3 |
| 2015 | Elbow | 37 | 2.74 |
| 2015 | Eye | 1 | 0.07 |
| 2015 | Face | 1 | 0.07 |
| 2015 | Fibula | 1 | 0.07 |
| 2015 | Finger | 16 | 1.19 |
| 2015 | Foot | 16 | 1.19 |
| 2015 | Forearm | 15 | 1.11 |
| 2015 | Hamstring | 44 | 3.26 |
| 2015 | Hand | 28 | 2.08 |
| 2015 | Head | 12 | 0.89 |
| 2015 | Heel | 6 | 0.44 |
| 2015 | Hip | 11 | 0.82 |
| 2015 | Intercostal | 1 | 0.07 |
| 2015 | Knee | 50 | 3.71 |
| 2015 | Leg | 12 | 0.89 |
| 2015 | Neck | 29 | 2.15 |
| 2015 | Oblique | 14 | 1.04 |
| 2015 | Quadricep | 10 | 0.74 |
| 2015 | Rib | 12 | 0.89 |
| 2015 | Shin | 4 | 0.3 |
| 2015 | Shoulder | 48 | 3.56 |
| 2015 | Thigh | 1 | 0.07 |
| 2015 | Thumb | 13 | 0.96 |
| 2015 | Toe | 3 | 0.22 |
| 2015 | Tricep | 2 | 0.15 |
| 2015 | Wrist | 32 | 2.37 |
| 2016 | Abdominal | 2 | 0.15 |
| 2016 | Achilles | 2 | 0.15 |
| 2016 | Adductor | 17 | 1.26 |
| 2016 | Ankle | 21 | 1.55 |
| 2016 | Arm | 7 | 0.52 |
| 2016 | Back | 39 | 2.88 |
| 2016 | Bicep | 4 | 0.3 |
| 2016 | Calf | 14 | 1.03 |
| 2016 | Concussion | 3 | 0.22 |
| 2016 | Elbow | 28 | 2.07 |
| 2016 | Eye | 3 | 0.22 |
| 2016 | Face | 2 | 0.15 |
| 2016 | Finger | 19 | 1.4 |
| 2016 | Foot | 25 | 1.85 |
| 2016 | Forearm | 14 | 1.03 |
| 2016 | Hamstring | 45 | 3.33 |
| 2016 | Hand | 21 | 1.55 |
| 2016 | Head | 12 | 0.89 |
| 2016 | Heel | 4 | 0.3 |
| 2016 | Hip | 13 | 0.96 |
| 2016 | Intercostal | 2 | 0.15 |
| 2016 | Knee | 36 | 2.66 |
| 2016 | Leg | 9 | 0.67 |
| 2016 | Neck | 23 | 1.7 |
| 2016 | Oblique | 14 | 1.03 |
| 2016 | Quadricep | 12 | 0.89 |
| 2016 | Rib | 7 | 0.52 |
| 2016 | Shin | 4 | 0.3 |
| 2016 | Shoulder | 35 | 2.59 |
| 2016 | Thigh | 1 | 0.07 |
| 2016 | Thumb | 22 | 1.63 |
| 2016 | Toe | 5 | 0.37 |
| 2016 | Tricep | 5 | 0.37 |
| 2016 | Wrist | 27 | 2 |
| 2017 | Abdominal | 3 | 0.22 |
| 2017 | Achilles | 2 | 0.15 |
| 2017 | Adductor | 24 | 1.77 |
| 2017 | Ankle | 22 | 1.62 |
| 2017 | Arm | 3 | 0.22 |
| 2017 | Back | 55 | 4.05 |
| 2017 | Bicep | 3 | 0.22 |
| 2017 | Calf | 12 | 0.88 |
| 2017 | Chest | 3 | 0.22 |
| 2017 | Collarbone | 1 | 0.07 |
| 2017 | Concussion | 5 | 0.37 |
| 2017 | Elbow | 18 | 1.33 |
| 2017 | Eye | 2 | 0.15 |
| 2017 | Face | 1 | 0.07 |
| 2017 | Fibula | 1 | 0.07 |
| 2017 | Finger | 18 | 1.33 |
| 2017 | Foot | 14 | 1.03 |
| 2017 | Forearm | 10 | 0.74 |
| 2017 | Hamstring | 48 | 3.53 |
| 2017 | Hand | 30 | 2.21 |
| 2017 | Head | 10 | 0.74 |
| 2017 | Heel | 2 | 0.15 |
| 2017 | Hernia | 1 | 0.07 |
| 2017 | Hip | 22 | 1.62 |
| 2017 | Intercostal | 2 | 0.15 |
| 2017 | Knee | 44 | 3.24 |
| 2017 | Leg | 5 | 0.37 |
| 2017 | Neck | 32 | 2.36 |
| 2017 | Oblique | 11 | 0.81 |
| 2017 | Quadricep | 9 | 0.66 |
| 2017 | Rib | 4 | 0.29 |
| 2017 | Shin | 2 | 0.15 |
| 2017 | Shoulder | 29 | 2.14 |
| 2017 | Thigh | 1 | 0.07 |
| 2017 | Thumb | 23 | 1.69 |
| 2017 | Toe | 3 | 0.22 |
| 2017 | Tricep | 2 | 0.15 |
| 2017 | Wrist | 23 | 1.69 |
| 2018 | Abdominal | 3 | 0.22 |
| 2018 | Achilles | 2 | 0.15 |
| 2018 | Adductor | 14 | 1.02 |
| 2018 | Ankle | 20 | 1.45 |
| 2018 | Back | 55 | 3.99 |
| 2018 | Bicep | 2 | 0.15 |
| 2018 | Calf | 14 | 1.02 |
| 2018 | Chest | 3 | 0.22 |
| 2018 | Concussion | 4 | 0.29 |
| 2018 | Elbow | 15 | 1.09 |
| 2018 | Eye | 1 | 0.07 |
| 2018 | Face | 1 | 0.07 |
| 2018 | Finger | 21 | 1.52 |
| 2018 | Foot | 18 | 1.31 |
| 2018 | Forearm | 10 | 0.73 |
| 2018 | Hamstring | 55 | 3.99 |
| 2018 | Hand | 16 | 1.16 |
| 2018 | Head | 14 | 1.02 |
| 2018 | Heel | 2 | 0.15 |
| 2018 | Hip | 18 | 1.31 |
| 2018 | Intercostal | 1 | 0.07 |
| 2018 | Jaw | 1 | 0.07 |
| 2018 | Knee | 48 | 3.48 |
| 2018 | Leg | 7 | 0.51 |
| 2018 | Neck | 11 | 0.8 |
| 2018 | Oblique | 11 | 0.8 |
| 2018 | Quadricep | 11 | 0.8 |
| 2018 | Rib | 6 | 0.44 |
| 2018 | Shin | 1 | 0.07 |
| 2018 | Shoulder | 27 | 1.96 |
| 2018 | Thigh | 1 | 0.07 |
| 2018 | Thumb | 8 | 0.58 |
| 2018 | Toe | 7 | 0.51 |
| 2018 | Tricep | 3 | 0.22 |
| 2018 | Wrist | 30 | 2.18 |
| 2019 | Abdominal | 7 | 0.5 |
| 2019 | Achilles | 2 | 0.14 |
| 2019 | Adductor | 22 | 1.56 |
| 2019 | Ankle | 30 | 2.13 |
| 2019 | Arm | 3 | 0.21 |
| 2019 | Back | 59 | 4.18 |
| 2019 | Bicep | 6 | 0.43 |
| 2019 | Calf | 16 | 1.13 |
| 2019 | Chest | 3 | 0.21 |
| 2019 | Concussion | 5 | 0.35 |
| 2019 | Elbow | 22 | 1.56 |
| 2019 | Eye | 2 | 0.14 |
| 2019 | Face | 3 | 0.21 |
| 2019 | Finger | 11 | 0.78 |
| 2019 | Foot | 19 | 1.35 |
| 2019 | Forearm | 9 | 0.64 |
| 2019 | Hamstring | 47 | 3.33 |
| 2019 | Hand | 29 | 2.06 |
| 2019 | Head | 11 | 0.78 |
| 2019 | Heel | 4 | 0.28 |
| 2019 | Hip | 15 | 1.06 |
| 2019 | Intercostal | 1 | 0.07 |
| 2019 | Jaw | 3 | 0.21 |
| 2019 | Knee | 48 | 3.4 |
| 2019 | Leg | 11 | 0.78 |
| 2019 | Neck | 15 | 1.06 |
| 2019 | Oblique | 20 | 1.42 |
| 2019 | Quadricep | 15 | 1.06 |
| 2019 | Rib | 4 | 0.28 |
| 2019 | Shin | 4 | 0.28 |
| 2019 | Shoulder | 42 | 2.98 |
| 2019 | Thumb | 15 | 1.06 |
| 2019 | Toe | 7 | 0.5 |
| 2019 | Tricep | 3 | 0.21 |
| 2019 | Wrist | 25 | 1.77 |

Appendix 6. Basketball Injury Incidence by Body Part per Season

| Season | Injured Body Part | Count | Seasonal Injury Incidence per 100 Players |
| --- | --- | --- | --- |
| 2007-2008 | Abdominal | 3 | 0.67 |
| 2007-2008 | Achilles | 5 | 1.11 |
| 2007-2008 | Adductor | 16 | 3.55 |
| 2007-2008 | Ankle | 59 | 13.08 |
| 2007-2008 | Arm | 1 | 0.22 |
| 2007-2008 | Back | 27 | 5.99 |
| 2007-2008 | Bicep | 1 | 0.22 |
| 2007-2008 | Calf | 7 | 1.55 |
| 2007-2008 | Chest | 1 | 0.22 |
| 2007-2008 | Concussion | 5 | 1.11 |
| 2007-2008 | Elbow | 6 | 1.33 |
| 2007-2008 | Eye | 2 | 0.44 |
| 2007-2008 | Finger | 7 | 1.55 |
| 2007-2008 | Foot | 15 | 3.33 |
| 2007-2008 | Hamstring | 8 | 1.77 |
| 2007-2008 | Hand | 4 | 0.89 |
| 2007-2008 | Head | 7 | 1.55 |
| 2007-2008 | Heel | 3 | 0.67 |
| 2007-2008 | Hernia | 1 | 0.22 |
| 2007-2008 | Hip | 10 | 2.22 |
| 2007-2008 | Knee | 52 | 11.53 |
| 2007-2008 | Leg | 5 | 1.11 |
| 2007-2008 | Neck | 1 | 0.22 |
| 2007-2008 | Patella | 2 | 0.44 |
| 2007-2008 | Quadricep | 8 | 1.77 |
| 2007-2008 | Rib | 2 | 0.44 |
| 2007-2008 | Shin | 1 | 0.22 |
| 2007-2008 | Shoulder | 10 | 2.22 |
| 2007-2008 | Thigh | 5 | 1.11 |
| 2007-2008 | Thumb | 8 | 1.77 |
| 2007-2008 | Toe | 3 | 0.67 |
| 2007-2008 | Wrist | 11 | 2.44 |
| 2008-2009 | Abdominal | 3 | 0.67 |
| 2008-2009 | Achilles | 5 | 1.12 |
| 2008-2009 | Adductor | 11 | 2.47 |
| 2008-2009 | Ankle | 61 | 13.71 |
| 2008-2009 | Arm | 1 | 0.22 |
| 2008-2009 | Back | 34 | 7.64 |
| 2008-2009 | Calf | 6 | 1.35 |
| 2008-2009 | Chest | 2 | 0.45 |
| 2008-2009 | Concussion | 7 | 1.57 |
| 2008-2009 | Elbow | 7 | 1.57 |
| 2008-2009 | Eye | 2 | 0.45 |
| 2008-2009 | Face | 1 | 0.22 |
| 2008-2009 | Fibula | 1 | 0.22 |
| 2008-2009 | Finger | 3 | 0.67 |
| 2008-2009 | Foot | 21 | 4.72 |
| 2008-2009 | Forearm | 1 | 0.22 |
| 2008-2009 | Hamstring | 14 | 3.15 |
| 2008-2009 | Hand | 3 | 0.67 |
| 2008-2009 | Head | 3 | 0.67 |
| 2008-2009 | Heel | 1 | 0.22 |
| 2008-2009 | Hernia | 2 | 0.45 |
| 2008-2009 | Hip | 12 | 2.7 |
| 2008-2009 | Jaw | 2 | 0.45 |
| 2008-2009 | Knee | 56 | 12.58 |
| 2008-2009 | Leg | 3 | 0.67 |
| 2008-2009 | Neck | 3 | 0.67 |
| 2008-2009 | Quadricep | 6 | 1.35 |
| 2008-2009 | Rib | 1 | 0.22 |
| 2008-2009 | Shin | 1 | 0.22 |
| 2008-2009 | Shoulder | 12 | 2.7 |
| 2008-2009 | Thigh | 3 | 0.67 |
| 2008-2009 | Thumb | 11 | 2.47 |
| 2008-2009 | Toe | 6 | 1.35 |
| 2008-2009 | Wrist | 12 | 2.7 |
| 2009-2010 | Abdominal | 3 | 0.68 |
| 2009-2010 | Achilles | 9 | 2.04 |
| 2009-2010 | Adductor | 8 | 1.81 |
| 2009-2010 | Ankle | 55 | 12.44 |
| 2009-2010 | Back | 39 | 8.82 |
| 2009-2010 | Calf | 7 | 1.58 |
| 2009-2010 | Concussion | 2 | 0.45 |
| 2009-2010 | Elbow | 3 | 0.68 |
| 2009-2010 | Eye | 3 | 0.68 |
| 2009-2010 | Face | 2 | 0.45 |
| 2009-2010 | Fibula | 1 | 0.23 |
| 2009-2010 | Finger | 10 | 2.26 |
| 2009-2010 | Foot | 14 | 3.17 |
| 2009-2010 | Forearm | 2 | 0.45 |
| 2009-2010 | Hamstring | 12 | 2.71 |
| 2009-2010 | Hand | 5 | 1.13 |
| 2009-2010 | Head | 5 | 1.13 |
| 2009-2010 | Heel | 2 | 0.45 |
| 2009-2010 | Hernia | 2 | 0.45 |
| 2009-2010 | Hip | 10 | 2.26 |
| 2009-2010 | Jaw | 4 | 0.9 |
| 2009-2010 | Knee | 55 | 12.44 |
| 2009-2010 | Neck | 2 | 0.45 |
| 2009-2010 | Oblique | 2 | 0.45 |
| 2009-2010 | Patella | 6 | 1.36 |
| 2009-2010 | Quadricep | 2 | 0.45 |
| 2009-2010 | Rib | 3 | 0.68 |
| 2009-2010 | Shoulder | 14 | 3.17 |
| 2009-2010 | Thigh | 3 | 0.68 |
| 2009-2010 | Thumb | 7 | 1.58 |
| 2009-2010 | Toe | 7 | 1.58 |
| 2009-2010 | Wrist | 7 | 1.58 |
| 2010-2011 | Abdominal | 1 | 0.22 |
| 2010-2011 | Abductor | 2 | 0.44 |
| 2010-2011 | Achilles | 4 | 0.88 |
| 2010-2011 | Adductor | 12 | 2.65 |
| 2010-2011 | Ankle | 53 | 11.73 |
| 2010-2011 | Arm | 1 | 0.22 |
| 2010-2011 | Back | 30 | 6.64 |
| 2010-2011 | Calf | 10 | 2.21 |
| 2010-2011 | Chest | 1 | 0.22 |
| 2010-2011 | Concussion | 5 | 1.11 |
| 2010-2011 | Elbow | 6 | 1.33 |
| 2010-2011 | Eye | 1 | 0.22 |
| 2010-2011 | Face | 1 | 0.22 |
| 2010-2011 | Fibula | 1 | 0.22 |
| 2010-2011 | Finger | 4 | 0.88 |
| 2010-2011 | Foot | 22 | 4.87 |
| 2010-2011 | Hamstring | 11 | 2.43 |
| 2010-2011 | Hand | 1 | 0.22 |
| 2010-2011 | Head | 4 | 0.88 |
| 2010-2011 | Heel | 1 | 0.22 |
| 2010-2011 | Hernia | 1 | 0.22 |
| 2010-2011 | Hip | 10 | 2.21 |
| 2010-2011 | Knee | 64 | 14.16 |
| 2010-2011 | Leg | 3 | 0.66 |
| 2010-2011 | Neck | 2 | 0.44 |
| 2010-2011 | Patella | 9 | 1.99 |
| 2010-2011 | Quadricep | 6 | 1.33 |
| 2010-2011 | Rib | 2 | 0.44 |
| 2010-2011 | Shin | 2 | 0.44 |
| 2010-2011 | Shoulder | 13 | 2.88 |
| 2010-2011 | Thigh | 3 | 0.66 |
| 2010-2011 | Thumb | 6 | 1.33 |
| 2010-2011 | Toe | 5 | 1.11 |
| 2010-2011 | Wrist | 8 | 1.77 |
| 2011-2012 | Abdominal | 1 | 0.21 |
| 2011-2012 | Abductor | 1 | 0.21 |
| 2011-2012 | Achilles | 8 | 1.67 |
| 2011-2012 | Adductor | 22 | 4.6 |
| 2011-2012 | Ankle | 63 | 13.18 |
| 2011-2012 | Back | 33 | 6.9 |
| 2011-2012 | Calf | 12 | 2.51 |
| 2011-2012 | Chest | 3 | 0.63 |
| 2011-2012 | Concussion | 13 | 2.72 |
| 2011-2012 | Elbow | 5 | 1.05 |
| 2011-2012 | Eye | 2 | 0.42 |
| 2011-2012 | Face | 2 | 0.42 |
| 2011-2012 | Finger | 5 | 1.05 |
| 2011-2012 | Foot | 23 | 4.81 |
| 2011-2012 | Hamstring | 16 | 3.35 |
| 2011-2012 | Hand | 7 | 1.46 |
| 2011-2012 | Head | 3 | 0.63 |
| 2011-2012 | Heel | 2 | 0.42 |
| 2011-2012 | Hernia | 2 | 0.42 |
| 2011-2012 | Hip | 11 | 2.3 |
| 2011-2012 | Knee | 59 | 12.34 |
| 2011-2012 | Leg | 1 | 0.21 |
| 2011-2012 | Oblique | 1 | 0.21 |
| 2011-2012 | Patella | 2 | 0.42 |
| 2011-2012 | Quadricep | 9 | 1.88 |
| 2011-2012 | Rib | 3 | 0.63 |
| 2011-2012 | Shin | 2 | 0.42 |
| 2011-2012 | Shoulder | 21 | 4.39 |
| 2011-2012 | Thigh | 5 | 1.05 |
| 2011-2012 | Thumb | 3 | 0.63 |
| 2011-2012 | Toe | 9 | 1.88 |
| 2011-2012 | Wrist | 10 | 2.09 |
| 2012-2013 | Abdominal | 6 | 1.28 |
| 2012-2013 | Abductor | 2 | 0.43 |
| 2012-2013 | Achilles | 4 | 0.85 |
| 2012-2013 | Adductor | 12 | 2.56 |
| 2012-2013 | Ankle | 84 | 17.91 |
| 2012-2013 | Arm | 1 | 0.21 |
| 2012-2013 | Back | 35 | 7.46 |
| 2012-2013 | Calf | 16 | 3.41 |
| 2012-2013 | Chest | 2 | 0.43 |
| 2012-2013 | Concussion | 9 | 1.92 |
| 2012-2013 | Elbow | 7 | 1.49 |
| 2012-2013 | Eye | 1 | 0.21 |
| 2012-2013 | Finger | 7 | 1.49 |
| 2012-2013 | Foot | 24 | 5.12 |
| 2012-2013 | Forearm | 1 | 0.21 |
| 2012-2013 | Hamstring | 16 | 3.41 |
| 2012-2013 | Hand | 8 | 1.71 |
| 2012-2013 | Head | 1 | 0.21 |
| 2012-2013 | Heel | 2 | 0.43 |
| 2012-2013 | Hernia | 1 | 0.21 |
| 2012-2013 | Hip | 13 | 2.77 |
| 2012-2013 | Knee | 73 | 15.57 |
| 2012-2013 | Leg | 5 | 1.07 |
| 2012-2013 | Neck | 7 | 1.49 |
| 2012-2013 | Patella | 1 | 0.21 |
| 2012-2013 | Quadricep | 9 | 1.92 |
| 2012-2013 | Rib | 2 | 0.43 |
| 2012-2013 | Shin | 2 | 0.43 |
| 2012-2013 | Shoulder | 19 | 4.05 |
| 2012-2013 | Thigh | 2 | 0.43 |
| 2012-2013 | Thumb | 6 | 1.28 |
| 2012-2013 | Toe | 3 | 0.64 |
| 2012-2013 | Tricep | 3 | 0.64 |
| 2012-2013 | Wrist | 4 | 0.85 |
| 2013-2014 | Abdominal | 3 | 0.62 |
| 2013-2014 | Achilles | 8 | 1.66 |
| 2013-2014 | Adductor | 10 | 2.07 |
| 2013-2014 | Ankle | 75 | 15.56 |
| 2013-2014 | Back | 38 | 7.88 |
| 2013-2014 | Bicep | 1 | 0.21 |
| 2013-2014 | Calf | 16 | 3.32 |
| 2013-2014 | Chest | 1 | 0.21 |
| 2013-2014 | Concussion | 2 | 0.41 |
| 2013-2014 | Elbow | 5 | 1.04 |
| 2013-2014 | Eye | 1 | 0.21 |
| 2013-2014 | Fibula | 1 | 0.21 |
| 2013-2014 | Finger | 4 | 0.83 |
| 2013-2014 | Foot | 24 | 4.98 |
| 2013-2014 | Hamstring | 17 | 3.53 |
| 2013-2014 | Hand | 10 | 2.07 |
| 2013-2014 | Head | 2 | 0.41 |
| 2013-2014 | Heel | 4 | 0.83 |
| 2013-2014 | Hernia | 1 | 0.21 |
| 2013-2014 | Hip | 10 | 2.07 |
| 2013-2014 | Knee | 84 | 17.43 |
| 2013-2014 | Leg | 2 | 0.41 |
| 2013-2014 | Neck | 5 | 1.04 |
| 2013-2014 | Patella | 6 | 1.24 |
| 2013-2014 | Quadricep | 4 | 0.83 |
| 2013-2014 | Rib | 7 | 1.45 |
| 2013-2014 | Shin | 3 | 0.62 |
| 2013-2014 | Shoulder | 17 | 3.53 |
| 2013-2014 | Thigh | 8 | 1.66 |
| 2013-2014 | Thumb | 4 | 0.83 |
| 2013-2014 | Toe | 8 | 1.66 |
| 2013-2014 | Wrist | 10 | 2.07 |
| 2014-2015 | Achilles | 12 | 2.44 |
| 2014-2015 | Adductor | 14 | 2.85 |
| 2014-2015 | Ankle | 82 | 16.67 |
| 2014-2015 | Back | 41 | 8.33 |
| 2014-2015 | Calf | 14 | 2.85 |
| 2014-2015 | Chest | 1 | 0.2 |
| 2014-2015 | Concussion | 10 | 2.03 |
| 2014-2015 | Elbow | 9 | 1.83 |
| 2014-2015 | Eye | 2 | 0.41 |
| 2014-2015 | Fibula | 1 | 0.2 |
| 2014-2015 | Finger | 4 | 0.81 |
| 2014-2015 | Foot | 15 | 3.05 |
| 2014-2015 | Forearm | 1 | 0.2 |
| 2014-2015 | Hamstring | 18 | 3.66 |
| 2014-2015 | Hand | 9 | 1.83 |
| 2014-2015 | Head | 3 | 0.61 |
| 2014-2015 | Heel | 3 | 0.61 |
| 2014-2015 | Hernia | 1 | 0.2 |
| 2014-2015 | Hip | 21 | 4.27 |
| 2014-2015 | Jaw | 1 | 0.2 |
| 2014-2015 | Knee | 64 | 13.01 |
| 2014-2015 | Leg | 8 | 1.63 |
| 2014-2015 | Neck | 3 | 0.61 |
| 2014-2015 | Oblique | 2 | 0.41 |
| 2014-2015 | Patella | 1 | 0.2 |
| 2014-2015 | Quadricep | 3 | 0.61 |
| 2014-2015 | Rib | 7 | 1.42 |
| 2014-2015 | Shin | 2 | 0.41 |
| 2014-2015 | Shoulder | 23 | 4.67 |
| 2014-2015 | Thigh | 4 | 0.81 |
| 2014-2015 | Thumb | 5 | 1.02 |
| 2014-2015 | Toe | 7 | 1.42 |
| 2014-2015 | Wrist | 11 | 2.24 |
| 2015-2016 | Abdominal | 4 | 0.84 |
| 2015-2016 | Abductor | 3 | 0.63 |
| 2015-2016 | Achilles | 9 | 1.89 |
| 2015-2016 | Adductor | 12 | 2.52 |
| 2015-2016 | Ankle | 71 | 14.92 |
| 2015-2016 | Back | 43 | 9.03 |
| 2015-2016 | Calf | 13 | 2.73 |
| 2015-2016 | Chest | 2 | 0.42 |
| 2015-2016 | Collarbone | 1 | 0.21 |
| 2015-2016 | Concussion | 14 | 2.94 |
| 2015-2016 | Elbow | 4 | 0.84 |
| 2015-2016 | Eye | 8 | 1.68 |
| 2015-2016 | Face | 1 | 0.21 |
| 2015-2016 | Fibula | 1 | 0.21 |
| 2015-2016 | Finger | 3 | 0.63 |
| 2015-2016 | Foot | 23 | 4.83 |
| 2015-2016 | Hamstring | 18 | 3.78 |
| 2015-2016 | Hand | 7 | 1.47 |
| 2015-2016 | Head | 5 | 1.05 |
| 2015-2016 | Heel | 3 | 0.63 |
| 2015-2016 | Hernia | 2 | 0.42 |
| 2015-2016 | Hip | 22 | 4.62 |
| 2015-2016 | Knee | 79 | 16.6 |
| 2015-2016 | Leg | 8 | 1.68 |
| 2015-2016 | Neck | 2 | 0.42 |
| 2015-2016 | Oblique | 1 | 0.21 |
| 2015-2016 | Patella | 1 | 0.21 |
| 2015-2016 | Quadricep | 9 | 1.89 |
| 2015-2016 | Rib | 4 | 0.84 |
| 2015-2016 | Shin | 2 | 0.42 |
| 2015-2016 | Shoulder | 28 | 5.88 |
| 2015-2016 | Thigh | 4 | 0.84 |
| 2015-2016 | Thumb | 6 | 1.26 |
| 2015-2016 | Toe | 12 | 2.52 |
| 2015-2016 | Tricep | 1 | 0.21 |
| 2015-2016 | Wrist | 9 | 1.89 |
| 2016-2017 | Achilles | 9 | 1.85 |
| 2016-2017 | Adductor | 13 | 2.67 |
| 2016-2017 | Ankle | 76 | 15.64 |
| 2016-2017 | Arm | 2 | 0.41 |
| 2016-2017 | Back | 24 | 4.94 |
| 2016-2017 | Bicep | 1 | 0.21 |
| 2016-2017 | Calf | 18 | 3.7 |
| 2016-2017 | Chest | 1 | 0.21 |
| 2016-2017 | Concussion | 11 | 2.26 |
| 2016-2017 | Elbow | 5 | 1.03 |
| 2016-2017 | Eye | 4 | 0.82 |
| 2016-2017 | Face | 1 | 0.21 |
| 2016-2017 | Fibula | 3 | 0.62 |
| 2016-2017 | Finger | 7 | 1.44 |
| 2016-2017 | Foot | 27 | 5.56 |
| 2016-2017 | Forearm | 1 | 0.21 |
| 2016-2017 | Hamstring | 23 | 4.73 |
| 2016-2017 | Hand | 8 | 1.65 |
| 2016-2017 | Head | 5 | 1.03 |
| 2016-2017 | Heel | 4 | 0.82 |
| 2016-2017 | Hip | 21 | 4.32 |
| 2016-2017 | Knee | 75 | 15.43 |
| 2016-2017 | Leg | 7 | 1.44 |
| 2016-2017 | Neck | 1 | 0.21 |
| 2016-2017 | Patella | 2 | 0.41 |
| 2016-2017 | Quadricep | 12 | 2.47 |
| 2016-2017 | Rib | 1 | 0.21 |
| 2016-2017 | Shoulder | 20 | 4.12 |
| 2016-2017 | Thigh | 1 | 0.21 |
| 2016-2017 | Thumb | 9 | 1.85 |
| 2016-2017 | Toe | 10 | 2.06 |
| 2016-2017 | Wrist | 15 | 3.09 |
| 2017-2018 | Abdominal | 2 | 0.37 |
| 2017-2018 | Achilles | 8 | 1.48 |
| 2017-2018 | Adductor | 12 | 2.22 |
| 2017-2018 | Ankle | 83 | 15.37 |
| 2017-2018 | Arm | 1 | 0.19 |
| 2017-2018 | Back | 29 | 5.37 |
| 2017-2018 | Calf | 10 | 1.85 |
| 2017-2018 | Concussion | 13 | 2.41 |
| 2017-2018 | Elbow | 8 | 1.48 |
| 2017-2018 | Eye | 4 | 0.74 |
| 2017-2018 | Face | 1 | 0.19 |
| 2017-2018 | Fibula | 2 | 0.37 |
| 2017-2018 | Finger | 3 | 0.56 |
| 2017-2018 | Foot | 20 | 3.7 |
| 2017-2018 | Gluteus | 1 | 0.19 |
| 2017-2018 | Hamstring | 14 | 2.59 |
| 2017-2018 | Hand | 9 | 1.67 |
| 2017-2018 | Head | 4 | 0.74 |
| 2017-2018 | Heel | 5 | 0.93 |
| 2017-2018 | Hernia | 1 | 0.19 |
| 2017-2018 | Hip | 17 | 3.15 |
| 2017-2018 | Jaw | 1 | 0.19 |
| 2017-2018 | Knee | 77 | 14.26 |
| 2017-2018 | Leg | 8 | 1.48 |
| 2017-2018 | Neck | 4 | 0.74 |
| 2017-2018 | Oblique | 1 | 0.19 |
| 2017-2018 | Patella | 5 | 0.93 |
| 2017-2018 | Quadricep | 7 | 1.3 |
| 2017-2018 | Rib | 4 | 0.74 |
| 2017-2018 | Shin | 1 | 0.19 |
| 2017-2018 | Shoulder | 22 | 4.07 |
| 2017-2018 | Thigh | 4 | 0.74 |
| 2017-2018 | Thumb | 5 | 0.93 |
| 2017-2018 | Toe | 8 | 1.48 |
| 2017-2018 | Tricep | 1 | 0.19 |
| 2017-2018 | Wrist | 12 | 2.22 |
| 2018-2019 | Abdominal | 3 | 0.57 |
| 2018-2019 | Achilles | 10 | 1.89 |
| 2018-2019 | Adductor | 15 | 2.83 |
| 2018-2019 | Ankle | 80 | 15.09 |
| 2018-2019 | Back | 37 | 6.98 |
| 2018-2019 | Calf | 8 | 1.51 |
| 2018-2019 | Collarbone | 1 | 0.19 |
| 2018-2019 | Concussion | 10 | 1.89 |
| 2018-2019 | Elbow | 7 | 1.32 |
| 2018-2019 | Eye | 2 | 0.38 |
| 2018-2019 | Finger | 5 | 0.94 |
| 2018-2019 | Foot | 22 | 4.15 |
| 2018-2019 | Hamstring | 19 | 3.58 |
| 2018-2019 | Hand | 7 | 1.32 |
| 2018-2019 | Head | 4 | 0.75 |
| 2018-2019 | Heel | 8 | 1.51 |
| 2018-2019 | Hip | 18 | 3.4 |
| 2018-2019 | Jaw | 1 | 0.19 |
| 2018-2019 | Knee | 79 | 14.91 |
| 2018-2019 | Leg | 4 | 0.75 |
| 2018-2019 | Neck | 7 | 1.32 |
| 2018-2019 | Oblique | 1 | 0.19 |
| 2018-2019 | Quadricep | 11 | 2.08 |
| 2018-2019 | Rib | 2 | 0.38 |
| 2018-2019 | Shin | 2 | 0.38 |
| 2018-2019 | Shoulder | 12 | 2.26 |
| 2018-2019 | Thigh | 5 | 0.94 |
| 2018-2019 | Thumb | 7 | 1.32 |
| 2018-2019 | Toe | 14 | 2.64 |
| 2018-2019 | Wrist | 5 | 0.94 |
| 2019-2020 | Achilles | 1 | 0.2 |
| 2019-2020 | Adductor | 5 | 1 |
| 2019-2020 | Ankle | 21 | 4.22 |
| 2019-2020 | Back | 10 | 2.01 |
| 2019-2020 | Calf | 4 | 0.8 |
| 2019-2020 | Concussion | 5 | 1 |
| 2019-2020 | Elbow | 2 | 0.4 |
| 2019-2020 | Eye | 1 | 0.2 |
| 2019-2020 | Fibula | 1 | 0.2 |
| 2019-2020 | Finger | 3 | 0.6 |
| 2019-2020 | Foot | 6 | 1.2 |
| 2019-2020 | Forearm | 1 | 0.2 |
| 2019-2020 | Hamstring | 13 | 2.61 |
| 2019-2020 | Hand | 4 | 0.8 |
| 2019-2020 | Head | 1 | 0.2 |
| 2019-2020 | Heel | 2 | 0.4 |
| 2019-2020 | Hip | 5 | 1 |
| 2019-2020 | Knee | 18 | 3.61 |
| 2019-2020 | Leg | 2 | 0.4 |
| 2019-2020 | Neck | 3 | 0.6 |
| 2019-2020 | Shoulder | 8 | 1.61 |
| 2019-2020 | Thigh | 2 | 0.4 |
| 2019-2020 | Thumb | 6 | 1.2 |
| 2019-2020 | Toe | 3 | 0.6 |
| 2019-2020 | Wrist | 4 | 0.8 |

Appendix 7. Football Injury Incidence by Body Part per Season

| Season | Injured Body Part | Count | Seasonal Injury Incidence per 100 Players |
| --- | --- | --- | --- |
| 2007-2008 | Achilles | 1 | 0.08 |
| 2007-2008 | Adductor | 12 | 0.91 |
| 2007-2008 | Ankle | 42 | 3.17 |
| 2007-2008 | Back | 13 | 0.98 |
| 2007-2008 | Bicep | 2 | 0.15 |
| 2007-2008 | Calf | 13 | 0.98 |
| 2007-2008 | Chest | 4 | 0.3 |
| 2007-2008 | Concussion | 14 | 1.06 |
| 2007-2008 | Elbow | 3 | 0.23 |
| 2007-2008 | Eye | 1 | 0.08 |
| 2007-2008 | Fibula | 3 | 0.23 |
| 2007-2008 | Finger | 1 | 0.08 |
| 2007-2008 | Foot | 11 | 0.83 |
| 2007-2008 | Forearm | 3 | 0.23 |
| 2007-2008 | Hamstring | 20 | 1.51 |
| 2007-2008 | Hand | 4 | 0.3 |
| 2007-2008 | Head | 3 | 0.23 |
| 2007-2008 | Hip | 3 | 0.23 |
| 2007-2008 | Knee | 74 | 5.59 |
| 2007-2008 | Neck | 9 | 0.68 |
| 2007-2008 | Quadricep | 11 | 0.83 |
| 2007-2008 | Rib | 2 | 0.15 |
| 2007-2008 | Shin | 1 | 0.08 |
| 2007-2008 | Shoulder | 13 | 0.98 |
| 2007-2008 | Thigh | 4 | 0.3 |
| 2007-2008 | Toe | 5 | 0.38 |
| 2007-2008 | Tricep | 1 | 0.08 |
| 2007-2008 | Wrist | 1 | 0.08 |
| 2008-2009 | Abdominal | 1 | 0.07 |
| 2008-2009 | Achilles | 3 | 0.22 |
| 2008-2009 | Adductor | 35 | 2.62 |
| 2008-2009 | Ankle | 52 | 3.9 |
| 2008-2009 | Arm | 4 | 0.3 |
| 2008-2009 | Back | 14 | 1.05 |
| 2008-2009 | Bicep | 2 | 0.15 |
| 2008-2009 | Calf | 11 | 0.82 |
| 2008-2009 | Chest | 7 | 0.52 |
| 2008-2009 | Concussion | 21 | 1.57 |
| 2008-2009 | Elbow | 8 | 0.6 |
| 2008-2009 | Face | 1 | 0.07 |
| 2008-2009 | Fibula | 1 | 0.07 |
| 2008-2009 | Finger | 4 | 0.3 |
| 2008-2009 | Foot | 18 | 1.35 |
| 2008-2009 | Forearm | 5 | 0.37 |
| 2008-2009 | Hamstring | 49 | 3.67 |
| 2008-2009 | Hand | 10 | 0.75 |
| 2008-2009 | Head | 11 | 0.82 |
| 2008-2009 | Heel | 2 | 0.15 |
| 2008-2009 | Hernia | 3 | 0.22 |
| 2008-2009 | Hip | 8 | 0.6 |
| 2008-2009 | Jaw | 3 | 0.22 |
| 2008-2009 | Knee | 99 | 7.42 |
| 2008-2009 | Leg | 7 | 0.52 |
| 2008-2009 | Neck | 11 | 0.82 |
| 2008-2009 | Quadricep | 5 | 0.37 |
| 2008-2009 | Rib | 6 | 0.45 |
| 2008-2009 | Shin | 1 | 0.07 |
| 2008-2009 | Shoulder | 36 | 2.7 |
| 2008-2009 | Thigh | 13 | 0.97 |
| 2008-2009 | Thumb | 4 | 0.3 |
| 2008-2009 | Toe | 7 | 0.52 |
| 2008-2009 | Tricep | 1 | 0.07 |
| 2008-2009 | Wrist | 5 | 0.37 |
| 2009-2010 | Adductor | 10 | 0.74 |
| 2009-2010 | Ankle | 49 | 3.65 |
| 2009-2010 | Back | 11 | 0.82 |
| 2009-2010 | Calf | 8 | 0.6 |
| 2009-2010 | Chest | 3 | 0.22 |
| 2009-2010 | Concussion | 17 | 1.27 |
| 2009-2010 | Elbow | 1 | 0.07 |
| 2009-2010 | Fibula | 2 | 0.15 |
| 2009-2010 | Finger | 5 | 0.37 |
| 2009-2010 | Foot | 16 | 1.19 |
| 2009-2010 | Forearm | 4 | 0.3 |
| 2009-2010 | Hamstring | 29 | 2.16 |
| 2009-2010 | Hand | 4 | 0.3 |
| 2009-2010 | Head | 5 | 0.37 |
| 2009-2010 | Hip | 4 | 0.3 |
| 2009-2010 | Knee | 79 | 5.88 |
| 2009-2010 | Neck | 4 | 0.3 |
| 2009-2010 | Quadricep | 5 | 0.37 |
| 2009-2010 | Rib | 2 | 0.15 |
| 2009-2010 | Shin | 1 | 0.07 |
| 2009-2010 | Shoulder | 7 | 0.52 |
| 2009-2010 | Thigh | 3 | 0.22 |
| 2009-2010 | Thumb | 2 | 0.15 |
| 2009-2010 | Toe | 4 | 0.3 |
| 2009-2010 | Tricep | 2 | 0.15 |
| 2009-2010 | Wrist | 4 | 0.3 |
| 2010-2011 | Achilles | 1 | 0.07 |
| 2010-2011 | Adductor | 16 | 1.16 |
| 2010-2011 | Ankle | 52 | 3.77 |
| 2010-2011 | Back | 8 | 0.58 |
| 2010-2011 | Bicep | 1 | 0.07 |
| 2010-2011 | Calf | 6 | 0.44 |
| 2010-2011 | Chest | 5 | 0.36 |
| 2010-2011 | Concussion | 28 | 2.03 |
| 2010-2011 | Elbow | 4 | 0.29 |
| 2010-2011 | Fibula | 2 | 0.15 |
| 2010-2011 | Finger | 3 | 0.22 |
| 2010-2011 | Foot | 14 | 1.02 |
| 2010-2011 | Forearm | 3 | 0.22 |
| 2010-2011 | Gluteus | 1 | 0.07 |
| 2010-2011 | Hamstring | 47 | 3.41 |
| 2010-2011 | Hand | 2 | 0.15 |
| 2010-2011 | Head | 14 | 1.02 |
| 2010-2011 | Heel | 1 | 0.07 |
| 2010-2011 | Hip | 4 | 0.29 |
| 2010-2011 | Knee | 71 | 5.15 |
| 2010-2011 | Leg | 1 | 0.07 |
| 2010-2011 | Neck | 8 | 0.58 |
| 2010-2011 | Quadricep | 6 | 0.44 |
| 2010-2011 | Rib | 3 | 0.22 |
| 2010-2011 | Shin | 1 | 0.07 |
| 2010-2011 | Shoulder | 14 | 1.02 |
| 2010-2011 | Thigh | 3 | 0.22 |
| 2010-2011 | Thumb | 2 | 0.15 |
| 2010-2011 | Toe | 6 | 0.44 |
| 2010-2011 | Tricep | 1 | 0.07 |
| 2010-2011 | Wrist | 3 | 0.22 |
| 2011-2012 | Abdominal | 2 | 0.15 |
| 2011-2012 | Achilles | 1 | 0.08 |
| 2011-2012 | Adductor | 17 | 1.29 |
| 2011-2012 | Ankle | 43 | 3.27 |
| 2011-2012 | Back | 12 | 0.91 |
| 2011-2012 | Bicep | 1 | 0.08 |
| 2011-2012 | Calf | 13 | 0.99 |
| 2011-2012 | Chest | 7 | 0.53 |
| 2011-2012 | Collarbone | 1 | 0.08 |
| 2011-2012 | Concussion | 27 | 2.06 |
| 2011-2012 | Elbow | 4 | 0.3 |
| 2011-2012 | Eye | 2 | 0.15 |
| 2011-2012 | Fibula | 3 | 0.23 |
| 2011-2012 | Finger | 2 | 0.15 |
| 2011-2012 | Foot | 24 | 1.83 |
| 2011-2012 | Forearm | 1 | 0.08 |
| 2011-2012 | Hamstring | 52 | 3.96 |
| 2011-2012 | Hand | 8 | 0.61 |
| 2011-2012 | Head | 15 | 1.14 |
| 2011-2012 | Heel | 1 | 0.08 |
| 2011-2012 | Hip | 4 | 0.3 |
| 2011-2012 | Jaw | 1 | 0.08 |
| 2011-2012 | Knee | 63 | 4.8 |
| 2011-2012 | Neck | 10 | 0.76 |
| 2011-2012 | Quadricep | 6 | 0.46 |
| 2011-2012 | Rib | 1 | 0.08 |
| 2011-2012 | Shoulder | 22 | 1.68 |
| 2011-2012 | Thigh | 4 | 0.3 |
| 2011-2012 | Thumb | 5 | 0.38 |
| 2011-2012 | Toe | 7 | 0.53 |
| 2011-2012 | Tricep | 1 | 0.08 |
| 2011-2012 | Wrist | 2 | 0.15 |
| 2012-2013 | Abdominal | 3 | 0.23 |
| 2012-2013 | Achilles | 1 | 0.08 |
| 2012-2013 | Adductor | 13 | 1.01 |
| 2012-2013 | Ankle | 66 | 5.12 |
| 2012-2013 | Back | 9 | 0.7 |
| 2012-2013 | Bicep | 1 | 0.08 |
| 2012-2013 | Calf | 14 | 1.09 |
| 2012-2013 | Chest | 3 | 0.23 |
| 2012-2013 | Collarbone | 2 | 0.16 |
| 2012-2013 | Concussion | 43 | 3.33 |
| 2012-2013 | Elbow | 2 | 0.16 |
| 2012-2013 | Finger | 1 | 0.08 |
| 2012-2013 | Foot | 19 | 1.47 |
| 2012-2013 | Forearm | 2 | 0.16 |
| 2012-2013 | Hamstring | 60 | 4.65 |
| 2012-2013 | Hand | 6 | 0.47 |
| 2012-2013 | Head | 12 | 0.93 |
| 2012-2013 | Hip | 4 | 0.31 |
| 2012-2013 | Knee | 76 | 5.89 |
| 2012-2013 | Neck | 6 | 0.47 |
| 2012-2013 | Quadricep | 3 | 0.23 |
| 2012-2013 | Rib | 8 | 0.62 |
| 2012-2013 | Shin | 1 | 0.08 |
| 2012-2013 | Shoulder | 22 | 1.71 |
| 2012-2013 | Thigh | 7 | 0.54 |
| 2012-2013 | Thumb | 2 | 0.16 |
| 2012-2013 | Toe | 5 | 0.39 |
| 2012-2013 | Wrist | 1 | 0.08 |
| 2013-2014 | Achilles | 2 | 0.15 |
| 2013-2014 | Adductor | 21 | 1.6 |
| 2013-2014 | Ankle | 52 | 3.96 |
| 2013-2014 | Back | 7 | 0.53 |
| 2013-2014 | Bicep | 1 | 0.08 |
| 2013-2014 | Calf | 7 | 0.53 |
| 2013-2014 | Chest | 4 | 0.3 |
| 2013-2014 | Collarbone | 1 | 0.08 |
| 2013-2014 | Concussion | 44 | 3.35 |
| 2013-2014 | Elbow | 5 | 0.38 |
| 2013-2014 | Fibula | 1 | 0.08 |
| 2013-2014 | Finger | 2 | 0.15 |
| 2013-2014 | Foot | 25 | 1.91 |
| 2013-2014 | Forearm | 1 | 0.08 |
| 2013-2014 | Hamstring | 43 | 3.28 |
| 2013-2014 | Hand | 5 | 0.38 |
| 2013-2014 | Head | 2 | 0.15 |
| 2013-2014 | Hip | 11 | 0.84 |
| 2013-2014 | Knee | 72 | 5.49 |
| 2013-2014 | Neck | 9 | 0.69 |
| 2013-2014 | Quadricep | 6 | 0.46 |
| 2013-2014 | Rib | 4 | 0.3 |
| 2013-2014 | Shoulder | 16 | 1.22 |
| 2013-2014 | Thigh | 4 | 0.3 |
| 2013-2014 | Thumb | 6 | 0.46 |
| 2013-2014 | Toe | 8 | 0.61 |
| 2013-2014 | Tricep | 1 | 0.08 |
| 2013-2014 | Wrist | 2 | 0.15 |
| 2014-2015 | Abdominal | 2 | 0.15 |
| 2014-2015 | Achilles | 1 | 0.07 |
| 2014-2015 | Adductor | 18 | 1.35 |
| 2014-2015 | Ankle | 77 | 5.76 |
| 2014-2015 | Back | 17 | 1.27 |
| 2014-2015 | Bicep | 2 | 0.15 |
| 2014-2015 | Calf | 15 | 1.12 |
| 2014-2015 | Chest | 4 | 0.3 |
| 2014-2015 | Collarbone | 2 | 0.15 |
| 2014-2015 | Concussion | 35 | 2.62 |
| 2014-2015 | Elbow | 4 | 0.3 |
| 2014-2015 | Fibula | 2 | 0.15 |
| 2014-2015 | Finger | 3 | 0.22 |
| 2014-2015 | Foot | 29 | 2.17 |
| 2014-2015 | Forearm | 3 | 0.22 |
| 2014-2015 | Hamstring | 62 | 4.64 |
| 2014-2015 | Hand | 10 | 0.75 |
| 2014-2015 | Head | 1 | 0.07 |
| 2014-2015 | Hernia | 1 | 0.07 |
| 2014-2015 | Hip | 5 | 0.37 |
| 2014-2015 | Knee | 83 | 6.21 |
| 2014-2015 | Neck | 1 | 0.07 |
| 2014-2015 | Oblique | 1 | 0.07 |
| 2014-2015 | Quadricep | 10 | 0.75 |
| 2014-2015 | Rib | 5 | 0.37 |
| 2014-2015 | Shin | 1 | 0.07 |
| 2014-2015 | Shoulder | 24 | 1.8 |
| 2014-2015 | Thigh | 6 | 0.45 |
| 2014-2015 | Thumb | 4 | 0.3 |
| 2014-2015 | Toe | 10 | 0.75 |
| 2014-2015 | Wrist | 4 | 0.3 |
| 2015-2016 | Abdominal | 5 | 0.38 |
| 2015-2016 | Achilles | 1 | 0.08 |
| 2015-2016 | Adductor | 19 | 1.44 |
| 2015-2016 | Ankle | 68 | 5.17 |
| 2015-2016 | Back | 11 | 0.84 |
| 2015-2016 | Bicep | 1 | 0.08 |
| 2015-2016 | Calf | 20 | 1.52 |
| 2015-2016 | Chest | 3 | 0.23 |
| 2015-2016 | Concussion | 88 | 6.69 |
| 2015-2016 | Elbow | 3 | 0.23 |
| 2015-2016 | Finger | 2 | 0.15 |
| 2015-2016 | Foot | 26 | 1.98 |
| 2015-2016 | Forearm | 2 | 0.15 |
| 2015-2016 | Hamstring | 51 | 3.88 |
| 2015-2016 | Hand | 9 | 0.68 |
| 2015-2016 | Hip | 7 | 0.53 |
| 2015-2016 | Knee | 93 | 7.07 |
| 2015-2016 | Neck | 12 | 0.91 |
| 2015-2016 | Quadricep | 7 | 0.53 |
| 2015-2016 | Rib | 10 | 0.76 |
| 2015-2016 | Shoulder | 38 | 2.89 |
| 2015-2016 | Thigh | 1 | 0.08 |
| 2015-2016 | Thumb | 2 | 0.15 |
| 2015-2016 | Toe | 8 | 0.61 |
| 2016-2017 | Abdominal | 1 | 0.08 |
| 2016-2017 | Achilles | 5 | 0.38 |
| 2016-2017 | Adductor | 22 | 1.66 |
| 2016-2017 | Ankle | 78 | 5.88 |
| 2016-2017 | Arm | 1 | 0.08 |
| 2016-2017 | Back | 18 | 1.36 |
| 2016-2017 | Bicep | 1 | 0.08 |
| 2016-2017 | Calf | 16 | 1.21 |
| 2016-2017 | Chest | 8 | 0.6 |
| 2016-2017 | Collarbone | 1 | 0.08 |
| 2016-2017 | Concussion | 85 | 6.41 |
| 2016-2017 | Elbow | 8 | 0.6 |
| 2016-2017 | Eye | 1 | 0.08 |
| 2016-2017 | Fibula | 3 | 0.23 |
| 2016-2017 | Foot | 25 | 1.88 |
| 2016-2017 | Forearm | 2 | 0.15 |
| 2016-2017 | Hamstring | 59 | 4.45 |
| 2016-2017 | Hand | 5 | 0.38 |
| 2016-2017 | Head | 1 | 0.08 |
| 2016-2017 | Hip | 4 | 0.3 |
| 2016-2017 | Knee | 78 | 5.88 |
| 2016-2017 | Leg | 1 | 0.08 |
| 2016-2017 | Neck | 8 | 0.6 |
| 2016-2017 | Quadricep | 10 | 0.75 |
| 2016-2017 | Rib | 5 | 0.38 |
| 2016-2017 | Shin | 2 | 0.15 |
| 2016-2017 | Shoulder | 27 | 2.03 |
| 2016-2017 | Thigh | 10 | 0.75 |
| 2016-2017 | Thumb | 2 | 0.15 |
| 2016-2017 | Toe | 7 | 0.53 |
| 2016-2017 | Tricep | 2 | 0.15 |
| 2016-2017 | Wrist | 1 | 0.08 |
| 2017-2018 | Abdominal | 4 | 0.3 |
| 2017-2018 | Achilles | 3 | 0.23 |
| 2017-2018 | Adductor | 21 | 1.59 |
| 2017-2018 | Ankle | 64 | 4.84 |
| 2017-2018 | Back | 22 | 1.66 |
| 2017-2018 | Bicep | 1 | 0.08 |
| 2017-2018 | Calf | 15 | 1.13 |
| 2017-2018 | Chest | 4 | 0.3 |
| 2017-2018 | Collarbone | 1 | 0.08 |
| 2017-2018 | Concussion | 86 | 6.5 |
| 2017-2018 | Elbow | 5 | 0.38 |
| 2017-2018 | Eye | 2 | 0.15 |
| 2017-2018 | Fibula | 1 | 0.08 |
| 2017-2018 | Foot | 17 | 1.28 |
| 2017-2018 | Forearm | 1 | 0.08 |
| 2017-2018 | Hamstring | 45 | 3.4 |
| 2017-2018 | Hand | 6 | 0.45 |
| 2017-2018 | Hip | 2 | 0.15 |
| 2017-2018 | Knee | 77 | 5.82 |
| 2017-2018 | Neck | 14 | 1.06 |
| 2017-2018 | Quadricep | 9 | 0.68 |
| 2017-2018 | Rib | 9 | 0.68 |
| 2017-2018 | Shin | 2 | 0.15 |
| 2017-2018 | Shoulder | 36 | 2.72 |
| 2017-2018 | Thigh | 5 | 0.38 |
| 2017-2018 | Thumb | 3 | 0.23 |
| 2017-2018 | Toe | 5 | 0.38 |
| 2017-2018 | Tricep | 2 | 0.15 |
| 2017-2018 | Wrist | 2 | 0.15 |
| 2018-2019 | Abdominal | 2 | 0.15 |
| 2018-2019 | Achilles | 4 | 0.3 |
| 2018-2019 | Adductor | 14 | 1.04 |
| 2018-2019 | Ankle | 58 | 4.31 |
| 2018-2019 | Back | 9 | 0.67 |
| 2018-2019 | Calf | 23 | 1.71 |
| 2018-2019 | Chest | 7 | 0.52 |
| 2018-2019 | Concussion | 43 | 3.19 |
| 2018-2019 | Elbow | 8 | 0.59 |
| 2018-2019 | Fibula | 2 | 0.15 |
| 2018-2019 | Foot | 27 | 2 |
| 2018-2019 | Forearm | 1 | 0.07 |
| 2018-2019 | Hamstring | 57 | 4.23 |
| 2018-2019 | Hand | 2 | 0.15 |
| 2018-2019 | Heel | 3 | 0.22 |
| 2018-2019 | Hip | 10 | 0.74 |
| 2018-2019 | Knee | 88 | 6.53 |
| 2018-2019 | Neck | 13 | 0.97 |
| 2018-2019 | Oblique | 1 | 0.07 |
| 2018-2019 | Quadricep | 6 | 0.45 |
| 2018-2019 | Rib | 4 | 0.3 |
| 2018-2019 | Shin | 1 | 0.07 |
| 2018-2019 | Shoulder | 28 | 2.08 |
| 2018-2019 | Thigh | 3 | 0.22 |
| 2018-2019 | Thumb | 4 | 0.3 |
| 2018-2019 | Toe | 10 | 0.74 |
| 2018-2019 | Wrist | 1 | 0.07 |
| 2019-2020 | Abdominal | 3 | 0.27 |
| 2019-2020 | Achilles | 4 | 0.36 |
| 2019-2020 | Adductor | 19 | 1.72 |
| 2019-2020 | Ankle | 55 | 4.97 |
| 2019-2020 | Back | 8 | 0.72 |
| 2019-2020 | Bicep | 1 | 0.09 |
| 2019-2020 | Calf | 7 | 0.63 |
| 2019-2020 | Chest | 7 | 0.63 |
| 2019-2020 | Collarbone | 1 | 0.09 |
| 2019-2020 | Concussion | 53 | 4.79 |
| 2019-2020 | Elbow | 5 | 0.45 |
| 2019-2020 | Fibula | 2 | 0.18 |
| 2019-2020 | Finger | 1 | 0.09 |
| 2019-2020 | Foot | 20 | 1.81 |
| 2019-2020 | Forearm | 3 | 0.27 |
| 2019-2020 | Hamstring | 55 | 4.97 |
| 2019-2020 | Hand | 7 | 0.63 |
| 2019-2020 | Hip | 6 | 0.54 |
| 2019-2020 | Jaw | 1 | 0.09 |
| 2019-2020 | Knee | 62 | 5.6 |
| 2019-2020 | Neck | 8 | 0.72 |
| 2019-2020 | Oblique | 2 | 0.18 |
| 2019-2020 | Quadricep | 10 | 0.9 |
| 2019-2020 | Rib | 5 | 0.45 |
| 2019-2020 | Shoulder | 15 | 1.36 |
| 2019-2020 | Thigh | 1 | 0.09 |
| 2019-2020 | Thumb | 2 | 0.18 |
| 2019-2020 | Toe | 4 | 0.36 |
| 2019-2020 | Tricep | 1 | 0.09 |
| 2019-2020 | Wrist | 2 | 0.18 |

Appendix 8. Hockey Injury Incidence by Body Part Per Season

| Season | Injured Body Part | Count | Seasonal Injury Incidence per 100 Players |
| --- | --- | --- | --- |
| 2007-2008 | Abdominal | 7 | 0.82 |
| 2007-2008 | Adductor | 71 | 8.33 |
| 2007-2008 | Ankle | 37 | 4.34 |
| 2007-2008 | Arm | 1 | 0.12 |
| 2007-2008 | Back | 40 | 4.69 |
| 2007-2008 | Calf | 3 | 0.35 |
| 2007-2008 | Chest | 5 | 0.59 |
| 2007-2008 | Concussion | 24 | 2.82 |
| 2007-2008 | Elbow | 2 | 0.23 |
| 2007-2008 | Eye | 6 | 0.7 |
| 2007-2008 | Face | 8 | 0.94 |
| 2007-2008 | Finger | 12 | 1.41 |
| 2007-2008 | Foot | 27 | 3.17 |
| 2007-2008 | Forearm | 1 | 0.12 |
| 2007-2008 | Gluteus | 1 | 0.12 |
| 2007-2008 | Hamstring | 7 | 0.82 |
| 2007-2008 | Hand | 16 | 1.88 |
| 2007-2008 | Head | 24 | 2.82 |
| 2007-2008 | Hernia | 4 | 0.47 |
| 2007-2008 | Hip | 29 | 3.4 |
| 2007-2008 | Jaw | 2 | 0.23 |
| 2007-2008 | Knee | 88 | 10.33 |
| 2007-2008 | Leg | 31 | 3.64 |
| 2007-2008 | Neck | 20 | 2.35 |
| 2007-2008 | Oblique | 3 | 0.35 |
| 2007-2008 | Quadricep | 2 | 0.23 |
| 2007-2008 | Rib | 6 | 0.7 |
| 2007-2008 | Shin | 1 | 0.12 |
| 2007-2008 | Shoulder | 64 | 7.51 |
| 2007-2008 | Thigh | 3 | 0.35 |
| 2007-2008 | Thumb | 3 | 0.35 |
| 2007-2008 | Toe | 3 | 0.35 |
| 2007-2008 | Wrist | 17 | 2 |
| 2008-2009 | Abdominal | 4 | 0.45 |
| 2008-2009 | Achilles | 1 | 0.11 |
| 2008-2009 | Adductor | 50 | 5.65 |
| 2008-2009 | Ankle | 26 | 2.94 |
| 2008-2009 | Arm | 11 | 1.24 |
| 2008-2009 | Back | 30 | 3.39 |
| 2008-2009 | Chest | 1 | 0.11 |
| 2008-2009 | Concussion | 26 | 2.94 |
| 2008-2009 | Elbow | 2 | 0.23 |
| 2008-2009 | Eye | 9 | 1.02 |
| 2008-2009 | Face | 8 | 0.9 |
| 2008-2009 | Finger | 12 | 1.36 |
| 2008-2009 | Foot | 32 | 3.62 |
| 2008-2009 | Hamstring | 3 | 0.34 |
| 2008-2009 | Hand | 20 | 2.26 |
| 2008-2009 | Head | 24 | 2.71 |
| 2008-2009 | Heel | 1 | 0.11 |
| 2008-2009 | Hernia | 1 | 0.11 |
| 2008-2009 | Hip | 17 | 1.92 |
| 2008-2009 | Jaw | 7 | 0.79 |
| 2008-2009 | Knee | 42 | 4.75 |
| 2008-2009 | Leg | 32 | 3.62 |
| 2008-2009 | Neck | 4 | 0.45 |
| 2008-2009 | Oblique | 1 | 0.11 |
| 2008-2009 | Rib | 8 | 0.9 |
| 2008-2009 | Shoulder | 39 | 4.41 |
| 2008-2009 | Thigh | 4 | 0.45 |
| 2008-2009 | Thumb | 1 | 0.11 |
| 2008-2009 | Toe | 1 | 0.11 |
| 2008-2009 | Wrist | 14 | 1.58 |
| 2009-2010 | Abdominal | 7 | 0.8 |
| 2009-2010 | Adductor | 45 | 5.12 |
| 2009-2010 | Ankle | 19 | 2.16 |
| 2009-2010 | Arm | 9 | 1.02 |
| 2009-2010 | Back | 28 | 3.19 |
| 2009-2010 | Chest | 2 | 0.23 |
| 2009-2010 | Concussion | 20 | 2.28 |
| 2009-2010 | Elbow | 4 | 0.46 |
| 2009-2010 | Eye | 7 | 0.8 |
| 2009-2010 | Face | 10 | 1.14 |
| 2009-2010 | Fibula | 1 | 0.11 |
| 2009-2010 | Finger | 10 | 1.14 |
| 2009-2010 | Foot | 40 | 4.55 |
| 2009-2010 | Forearm | 1 | 0.11 |
| 2009-2010 | Hamstring | 3 | 0.34 |
| 2009-2010 | Hand | 17 | 1.93 |
| 2009-2010 | Head | 40 | 4.55 |
| 2009-2010 | Hernia | 1 | 0.11 |
| 2009-2010 | Hip | 13 | 1.48 |
| 2009-2010 | Jaw | 2 | 0.23 |
| 2009-2010 | Knee | 61 | 6.94 |
| 2009-2010 | Leg | 34 | 3.87 |
| 2009-2010 | Neck | 7 | 0.8 |
| 2009-2010 | Oblique | 2 | 0.23 |
| 2009-2010 | Patella | 1 | 0.11 |
| 2009-2010 | Quadricep | 1 | 0.11 |
| 2009-2010 | Rib | 8 | 0.91 |
| 2009-2010 | Shoulder | 32 | 3.64 |
| 2009-2010 | Thigh | 1 | 0.11 |
| 2009-2010 | Thumb | 1 | 0.11 |
| 2009-2010 | Toe | 1 | 0.11 |
| 2009-2010 | Wrist | 7 | 0.8 |
| 2010-2011 | Abdominal | 1 | 0.11 |
| 2010-2011 | Adductor | 46 | 5.16 |
| 2010-2011 | Ankle | 23 | 2.58 |
| 2010-2011 | Arm | 5 | 0.56 |
| 2010-2011 | Back | 23 | 2.58 |
| 2010-2011 | Calf | 1 | 0.11 |
| 2010-2011 | Collarbone | 1 | 0.11 |
| 2010-2011 | Concussion | 40 | 4.49 |
| 2010-2011 | Elbow | 2 | 0.22 |
| 2010-2011 | Eye | 4 | 0.45 |
| 2010-2011 | Face | 10 | 1.12 |
| 2010-2011 | Finger | 10 | 1.12 |
| 2010-2011 | Foot | 22 | 2.47 |
| 2010-2011 | Forearm | 3 | 0.34 |
| 2010-2011 | Hamstring | 4 | 0.45 |
| 2010-2011 | Hand | 19 | 2.13 |
| 2010-2011 | Head | 31 | 3.48 |
| 2010-2011 | Hernia | 1 | 0.11 |
| 2010-2011 | Hip | 13 | 1.46 |
| 2010-2011 | Jaw | 3 | 0.34 |
| 2010-2011 | Knee | 52 | 5.84 |
| 2010-2011 | Leg | 20 | 2.24 |
| 2010-2011 | Neck | 12 | 1.35 |
| 2010-2011 | Oblique | 1 | 0.11 |
| 2010-2011 | Quadricep | 1 | 0.11 |
| 2010-2011 | Rib | 8 | 0.9 |
| 2010-2011 | Shoulder | 33 | 3.7 |
| 2010-2011 | Thigh | 2 | 0.22 |
| 2010-2011 | Thumb | 3 | 0.34 |
| 2010-2011 | Toe | 1 | 0.11 |
| 2010-2011 | Wrist | 6 | 0.67 |
| 2011-2012 | Abdominal | 1 | 0.11 |
| 2011-2012 | Achilles | 1 | 0.11 |
| 2011-2012 | Adductor | 50 | 5.59 |
| 2011-2012 | Ankle | 21 | 2.35 |
| 2011-2012 | Arm | 6 | 0.67 |
| 2011-2012 | Back | 34 | 3.8 |
| 2011-2012 | Bicep | 1 | 0.11 |
| 2011-2012 | Chest | 1 | 0.11 |
| 2011-2012 | Collarbone | 2 | 0.22 |
| 2011-2012 | Concussion | 49 | 5.48 |
| 2011-2012 | Elbow | 1 | 0.11 |
| 2011-2012 | Eye | 4 | 0.45 |
| 2011-2012 | Face | 15 | 1.68 |
| 2011-2012 | Finger | 5 | 0.56 |
| 2011-2012 | Foot | 24 | 2.68 |
| 2011-2012 | Forearm | 1 | 0.11 |
| 2011-2012 | Hamstring | 1 | 0.11 |
| 2011-2012 | Hand | 24 | 2.68 |
| 2011-2012 | Head | 30 | 3.36 |
| 2011-2012 | Hernia | 1 | 0.11 |
| 2011-2012 | Hip | 10 | 1.12 |
| 2011-2012 | Jaw | 2 | 0.22 |
| 2011-2012 | Knee | 34 | 3.8 |
| 2011-2012 | Leg | 25 | 2.8 |
| 2011-2012 | Neck | 3 | 0.34 |
| 2011-2012 | Quadricep | 2 | 0.22 |
| 2011-2012 | Rib | 1 | 0.11 |
| 2011-2012 | Shin | 1 | 0.11 |
| 2011-2012 | Shoulder | 23 | 2.57 |
| 2011-2012 | Thigh | 3 | 0.34 |
| 2011-2012 | Thumb | 3 | 0.34 |
| 2011-2012 | Wrist | 12 | 1.34 |
| 2012-2013 | Adductor | 15 | 1.79 |
| 2012-2013 | Ankle | 12 | 1.43 |
| 2012-2013 | Arm | 1 | 0.12 |
| 2012-2013 | Back | 11 | 1.31 |
| 2012-2013 | Bicep | 1 | 0.12 |
| 2012-2013 | Chest | 2 | 0.24 |
| 2012-2013 | Collarbone | 1 | 0.12 |
| 2012-2013 | Concussion | 25 | 2.98 |
| 2012-2013 | Elbow | 2 | 0.24 |
| 2012-2013 | Eye | 3 | 0.36 |
| 2012-2013 | Face | 5 | 0.6 |
| 2012-2013 | Fibula | 1 | 0.12 |
| 2012-2013 | Finger | 4 | 0.48 |
| 2012-2013 | Foot | 11 | 1.31 |
| 2012-2013 | Hamstring | 1 | 0.12 |
| 2012-2013 | Hand | 11 | 1.31 |
| 2012-2013 | Head | 18 | 2.15 |
| 2012-2013 | Heel | 1 | 0.12 |
| 2012-2013 | Hip | 8 | 0.95 |
| 2012-2013 | Jaw | 2 | 0.24 |
| 2012-2013 | Knee | 16 | 1.91 |
| 2012-2013 | Leg | 11 | 1.31 |
| 2012-2013 | Neck | 4 | 0.48 |
| 2012-2013 | Shoulder | 17 | 2.03 |
| 2012-2013 | Thigh | 2 | 0.24 |
| 2012-2013 | Thumb | 1 | 0.12 |
| 2012-2013 | Wrist | 7 | 0.83 |
| 2013-2014 | Abdominal | 3 | 0.36 |
| 2013-2014 | Adductor | 23 | 2.74 |
| 2013-2014 | Ankle | 21 | 2.5 |
| 2013-2014 | Arm | 3 | 0.36 |
| 2013-2014 | Back | 18 | 2.15 |
| 2013-2014 | Chest | 1 | 0.12 |
| 2013-2014 | Collarbone | 1 | 0.12 |
| 2013-2014 | Concussion | 36 | 4.29 |
| 2013-2014 | Elbow | 3 | 0.36 |
| 2013-2014 | Eye | 3 | 0.36 |
| 2013-2014 | Face | 11 | 1.31 |
| 2013-2014 | Fibula | 1 | 0.12 |
| 2013-2014 | Finger | 2 | 0.24 |
| 2013-2014 | Foot | 22 | 2.62 |
| 2013-2014 | Hamstring | 3 | 0.36 |
| 2013-2014 | Hand | 20 | 2.38 |
| 2013-2014 | Head | 18 | 2.15 |
| 2013-2014 | Heel | 1 | 0.12 |
| 2013-2014 | Hernia | 3 | 0.36 |
| 2013-2014 | Hip | 3 | 0.36 |
| 2013-2014 | Jaw | 3 | 0.36 |
| 2013-2014 | Knee | 40 | 4.77 |
| 2013-2014 | Leg | 21 | 2.5 |
| 2013-2014 | Neck | 11 | 1.31 |
| 2013-2014 | Oblique | 2 | 0.24 |
| 2013-2014 | Rib | 4 | 0.48 |
| 2013-2014 | Shoulder | 15 | 1.79 |
| 2013-2014 | Thumb | 2 | 0.24 |
| 2013-2014 | Wrist | 10 | 1.19 |
| 2014-2015 | Abdominal | 1 | 0.11 |
| 2014-2015 | Adductor | 11 | 1.25 |
| 2014-2015 | Ankle | 6 | 0.68 |
| 2014-2015 | Arm | 4 | 0.45 |
| 2014-2015 | Back | 10 | 1.13 |
| 2014-2015 | Concussion | 22 | 2.49 |
| 2014-2015 | Elbow | 1 | 0.11 |
| 2014-2015 | Eye | 4 | 0.45 |
| 2014-2015 | Face | 9 | 1.02 |
| 2014-2015 | Finger | 4 | 0.45 |
| 2014-2015 | Foot | 13 | 1.47 |
| 2014-2015 | Hamstring | 3 | 0.34 |
| 2014-2015 | Hand | 13 | 1.47 |
| 2014-2015 | Head | 14 | 1.59 |
| 2014-2015 | Hernia | 1 | 0.11 |
| 2014-2015 | Hip | 3 | 0.34 |
| 2014-2015 | Jaw | 3 | 0.34 |
| 2014-2015 | Knee | 12 | 1.36 |
| 2014-2015 | Leg | 16 | 1.81 |
| 2014-2015 | Neck | 2 | 0.23 |
| 2014-2015 | Oblique | 2 | 0.23 |
| 2014-2015 | Rib | 2 | 0.23 |
| 2014-2015 | Shoulder | 15 | 1.7 |
| 2014-2015 | Thumb | 1 | 0.11 |
| 2014-2015 | Wrist | 3 | 0.34 |
| 2015-2016 | Achilles | 1 | 0.11 |
| 2015-2016 | Adductor | 11 | 1.22 |
| 2015-2016 | Ankle | 7 | 0.78 |
| 2015-2016 | Arm | 4 | 0.45 |
| 2015-2016 | Back | 8 | 0.89 |
| 2015-2016 | Chest | 1 | 0.11 |
| 2015-2016 | Concussion | 22 | 2.45 |
| 2015-2016 | Elbow | 1 | 0.11 |
| 2015-2016 | Eye | 2 | 0.22 |
| 2015-2016 | Face | 1 | 0.11 |
| 2015-2016 | Fibula | 2 | 0.22 |
| 2015-2016 | Finger | 2 | 0.22 |
| 2015-2016 | Foot | 12 | 1.34 |
| 2015-2016 | Hand | 14 | 1.56 |
| 2015-2016 | Head | 9 | 1 |
| 2015-2016 | Hernia | 3 | 0.33 |
| 2015-2016 | Hip | 4 | 0.45 |
| 2015-2016 | Jaw | 3 | 0.33 |
| 2015-2016 | Knee | 23 | 2.56 |
| 2015-2016 | Leg | 5 | 0.56 |
| 2015-2016 | Neck | 5 | 0.56 |
| 2015-2016 | Oblique | 5 | 0.56 |
| 2015-2016 | Rib | 2 | 0.22 |
| 2015-2016 | Shoulder | 8 | 0.89 |
| 2015-2016 | Thumb | 2 | 0.22 |
| 2015-2016 | Tricep | 1 | 0.11 |
| 2015-2016 | Wrist | 4 | 0.45 |
| 2016-2017 | Abdominal | 1 | 0.11 |
| 2016-2017 | Adductor | 11 | 1.24 |
| 2016-2017 | Ankle | 10 | 1.13 |
| 2016-2017 | Back | 6 | 0.68 |
| 2016-2017 | Calf | 1 | 0.11 |
| 2016-2017 | Concussion | 23 | 2.59 |
| 2016-2017 | Elbow | 2 | 0.23 |
| 2016-2017 | Eye | 3 | 0.34 |
| 2016-2017 | Face | 5 | 0.56 |
| 2016-2017 | Fibula | 1 | 0.11 |
| 2016-2017 | Finger | 8 | 0.9 |
| 2016-2017 | Foot | 14 | 1.58 |
| 2016-2017 | Hamstring | 1 | 0.11 |
| 2016-2017 | Hand | 9 | 1.01 |
| 2016-2017 | Head | 4 | 0.45 |
| 2016-2017 | Hernia | 1 | 0.11 |
| 2016-2017 | Hip | 11 | 1.24 |
| 2016-2017 | Knee | 22 | 2.48 |
| 2016-2017 | Leg | 8 | 0.9 |
| 2016-2017 | Neck | 8 | 0.9 |
| 2016-2017 | Oblique | 1 | 0.11 |
| 2016-2017 | Rib | 3 | 0.34 |
| 2016-2017 | Shoulder | 9 | 1.01 |
| 2016-2017 | Thigh | 1 | 0.11 |
| 2016-2017 | Wrist | 5 | 0.56 |
| 2017-2018 | Abdominal | 2 | 0.22 |
| 2017-2018 | Adductor | 12 | 1.35 |
| 2017-2018 | Ankle | 10 | 1.12 |
| 2017-2018 | Arm | 2 | 0.22 |
| 2017-2018 | Back | 11 | 1.24 |
| 2017-2018 | Bicep | 1 | 0.11 |
| 2017-2018 | Concussion | 28 | 3.15 |
| 2017-2018 | Elbow | 2 | 0.22 |
| 2017-2018 | Eye | 1 | 0.11 |
| 2017-2018 | Face | 7 | 0.79 |
| 2017-2018 | Finger | 2 | 0.22 |
| 2017-2018 | Foot | 13 | 1.46 |
| 2017-2018 | Hamstring | 1 | 0.11 |
| 2017-2018 | Hand | 16 | 1.8 |
| 2017-2018 | Head | 8 | 0.9 |
| 2017-2018 | Hip | 6 | 0.67 |
| 2017-2018 | Jaw | 3 | 0.34 |
| 2017-2018 | Knee | 18 | 2.02 |
| 2017-2018 | Leg | 10 | 1.12 |
| 2017-2018 | Neck | 7 | 0.79 |
| 2017-2018 | Quadricep | 1 | 0.11 |
| 2017-2018 | Rib | 2 | 0.22 |
| 2017-2018 | Shoulder | 12 | 1.35 |
| 2017-2018 | Thumb | 2 | 0.22 |
| 2017-2018 | Wrist | 2 | 0.22 |
| 2018-2019 | Abdominal | 1 | 0.11 |
| 2018-2019 | Adductor | 8 | 0.88 |
| 2018-2019 | Ankle | 9 | 0.99 |
| 2018-2019 | Arm | 5 | 0.55 |
| 2018-2019 | Back | 12 | 1.32 |
| 2018-2019 | Concussion | 40 | 4.42 |
| 2018-2019 | Eye | 5 | 0.55 |
| 2018-2019 | Face | 3 | 0.33 |
| 2018-2019 | Fibula | 1 | 0.11 |
| 2018-2019 | Finger | 3 | 0.33 |
| 2018-2019 | Foot | 12 | 1.32 |
| 2018-2019 | Forearm | 1 | 0.11 |
| 2018-2019 | Hamstring | 2 | 0.22 |
| 2018-2019 | Hand | 12 | 1.32 |
| 2018-2019 | Head | 7 | 0.77 |
| 2018-2019 | Hip | 5 | 0.55 |
| 2018-2019 | Jaw | 1 | 0.11 |
| 2018-2019 | Knee | 15 | 1.66 |
| 2018-2019 | Leg | 10 | 1.1 |
| 2018-2019 | Neck | 6 | 0.66 |
| 2018-2019 | Oblique | 2 | 0.22 |
| 2018-2019 | Shoulder | 12 | 1.32 |
| 2018-2019 | Thumb | 1 | 0.11 |
| 2018-2019 | Wrist | 4 | 0.44 |
| 2019-2020 | Adductor | 3 | 0.36 |
| 2019-2020 | Ankle | 3 | 0.36 |
| 2019-2020 | Back | 2 | 0.24 |
| 2019-2020 | Concussion | 7 | 0.83 |
| 2019-2020 | Face | 2 | 0.24 |
| 2019-2020 | Finger | 2 | 0.24 |
| 2019-2020 | Foot | 2 | 0.24 |
| 2019-2020 | Hand | 4 | 0.47 |
| 2019-2020 | Head | 1 | 0.12 |
| 2019-2020 | Jaw | 1 | 0.12 |
| 2019-2020 | Knee | 6 | 0.71 |
| 2019-2020 | Leg | 8 | 0.95 |
| 2019-2020 | Rib | 1 | 0.12 |
| 2019-2020 | Shoulder | 5 | 0.59 |
| 2019-2020 | Thumb | 1 | 0.12 |
| 2019-2020 | Wrist | 1 | 0.12 |

Appendix 9. Five Year Knee Injury Incidence


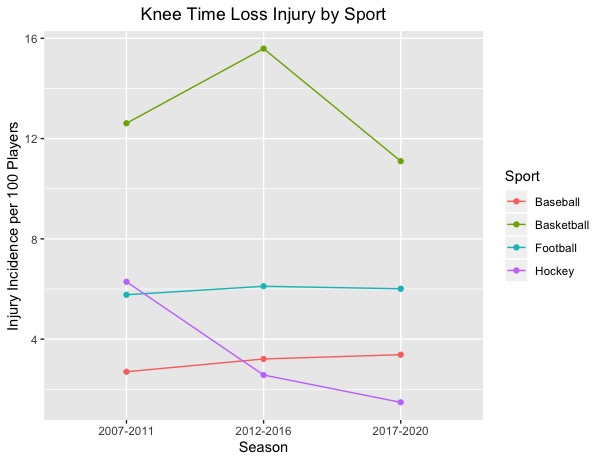


Appendix 10. Five Year Ankle Injury Incidence


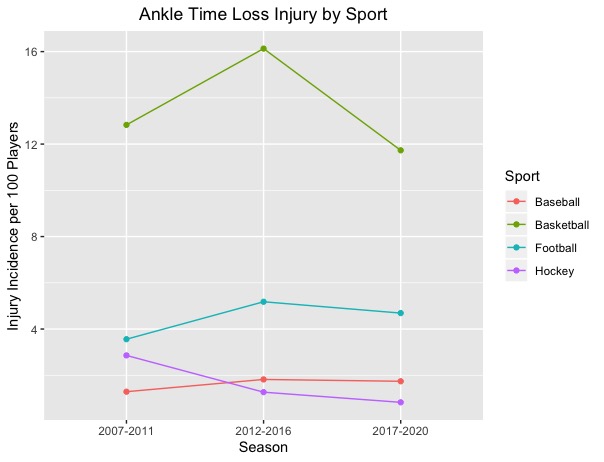


Appendix 11. Five Year Back Injury Incidence


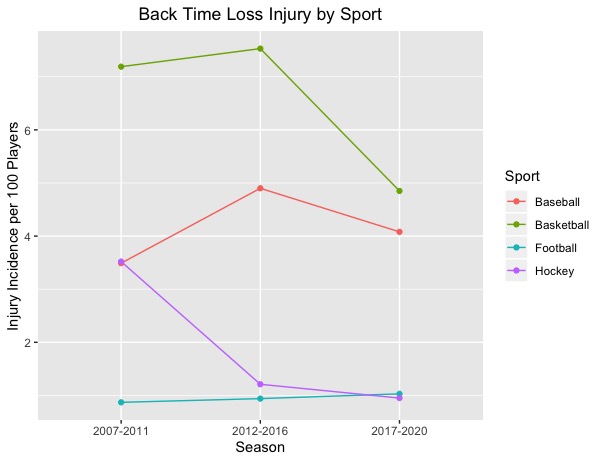


Appendix 12. Five Year Concussion Incidence


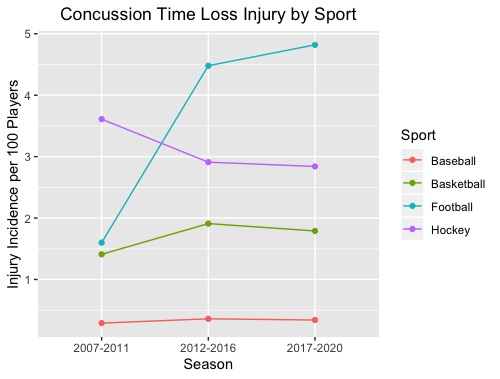


Appendix 13. Baseball Five Year Injury Incidence by Body Part

| Season | Body Part Injured | Count | Five Year Proportional Injury Incidence per 100 Players |
| --- | --- | --- | --- |
| 2007-2011 | Abdominal | 27 | 0.3 |
| 2007-2011 | Abductor | 1 | 0.01 |
| 2007-2011 | Achilles | 12 | 0.14 |
| 2007-2011 | Adductor | 120 | 1.35 |
| 2007-2011 | Ankle | 114 | 1.29 |
| 2007-2011 | Arm | 28 | 0.32 |
| 2007-2011 | Back | 309 | 3.49 |
| 2007-2011 | Bicep | 19 | 0.21 |
| 2007-2011 | Calf | 80 | 0.9 |
| 2007-2011 | Chest | 12 | 0.14 |
| 2007-2011 | Collarbone | 2 | 0.02 |
| 2007-2011 | Concussion | 26 | 0.29 |
| 2007-2011 | Elbow | 186 | 2.1 |
| 2007-2011 | Eye | 25 | 0.28 |
| 2007-2011 | Face | 8 | 0.09 |
| 2007-2011 | Finger | 110 | 1.24 |
| 2007-2011 | Foot | 73 | 0.82 |
| 2007-2011 | Forearm | 66 | 0.75 |
| 2007-2011 | Gluteus | 5 | 0.06 |
| 2007-2011 | Hamstring | 245 | 2.77 |
| 2007-2011 | Hand | 94 | 1.06 |
| 2007-2011 | Head | 51 | 0.58 |
| 2007-2011 | Heel | 20 | 0.23 |
| 2007-2011 | Hernia | 11 | 0.12 |
| 2007-2011 | Hip | 71 | 0.8 |
| 2007-2011 | Intercostal | 2 | 0.02 |
| 2007-2011 | Jaw | 2 | 0.02 |
| 2007-2011 | Knee | 239 | 2.7 |
| 2007-2011 | Leg | 41 | 0.46 |
| 2007-2011 | Neck | 95 | 1.07 |
| 2007-2011 | Oblique | 88 | 0.99 |
| 2007-2011 | Patella | 1 | 0.01 |
| 2007-2011 | Quadricep | 78 | 0.88 |
| 2007-2011 | Rib | 49 | 0.55 |
| 2007-2011 | Shin | 23 | 0.26 |
| 2007-2011 | Shoulder | 248 | 2.8 |
| 2007-2011 | Thigh | 10 | 0.11 |
| 2007-2011 | Thumb | 80 | 0.9 |
| 2007-2011 | Toe | 31 | 0.35 |
| 2007-2011 | Tricep | 18 | 0.2 |
| 2007-2011 | Wrist | 125 | 1.41 |
| 2012-2016 | Abdominal | 23 | 0.35 |
| 2012-2016 | Abductor | 1 | 0.02 |
| 2012-2016 | Achilles | 19 | 0.29 |
| 2012-2016 | Adductor | 123 | 1.86 |
| 2012-2016 | Ankle | 120 | 1.82 |
| 2012-2016 | Arm | 29 | 0.44 |
| 2012-2016 | Back | 324 | 4.9 |
| 2012-2016 | Bicep | 23 | 0.35 |
| 2012-2016 | Calf | 77 | 1.16 |
| 2012-2016 | Chest | 9 | 0.14 |
| 2012-2016 | Collarbone | 2 | 0.03 |
| 2012-2016 | Concussion | 24 | 0.36 |
| 2012-2016 | Elbow | 199 | 3.01 |
| 2012-2016 | Eye | 16 | 0.24 |
| 2012-2016 | Face | 11 | 0.17 |
| 2012-2016 | Fibula | 3 | 0.05 |
| 2012-2016 | Finger | 94 | 1.42 |
| 2012-2016 | Foot | 95 | 1.44 |
| 2012-2016 | Forearm | 63 | 0.95 |
| 2012-2016 | Hamstring | 262 | 3.96 |
| 2012-2016 | Hand | 136 | 2.06 |
| 2012-2016 | Head | 64 | 0.97 |
| 2012-2016 | Heel | 17 | 0.26 |
| 2012-2016 | Hernia | 3 | 0.05 |
| 2012-2016 | Hip | 60 | 0.91 |
| 2012-2016 | Intercostal | 7 | 0.11 |
| 2012-2016 | Jaw | 4 | 0.06 |
| 2012-2016 | Knee | 212 | 3.21 |
| 2012-2016 | Leg | 48 | 0.73 |
| 2012-2016 | Neck | 111 | 1.68 |
| 2012-2016 | Oblique | 93 | 1.41 |
| 2012-2016 | Quadricep | 73 | 1.1 |
| 2012-2016 | Rib | 44 | 0.67 |
| 2012-2016 | Shin | 27 | 0.41 |
| 2012-2016 | Shoulder | 237 | 3.59 |
| 2012-2016 | Thigh | 5 | 0.08 |
| 2012-2016 | Thumb | 85 | 1.29 |
| 2012-2016 | Toe | 26 | 0.39 |
| 2012-2016 | Tricep | 20 | 0.3 |
| 2012-2016 | Wrist | 148 | 2.24 |
| 2017-2020 | Abdominal | 13 | 0.31 |
| 2017-2020 | Achilles | 6 | 0.14 |
| 2017-2020 | Adductor | 60 | 1.45 |
| 2017-2020 | Ankle | 72 | 1.74 |
| 2017-2020 | Arm | 6 | 0.14 |
| 2017-2020 | Back | 169 | 4.08 |
| 2017-2020 | Bicep | 11 | 0.27 |
| 2017-2020 | Calf | 42 | 1.01 |
| 2017-2020 | Chest | 9 | 0.22 |
| 2017-2020 | Collarbone | 1 | 0.02 |
| 2017-2020 | Concussion | 14 | 0.34 |
| 2017-2020 | Elbow | 55 | 1.33 |
| 2017-2020 | Eye | 5 | 0.12 |
| 2017-2020 | Face | 5 | 0.12 |
| 2017-2020 | Fibula | 1 | 0.02 |
| 2017-2020 | Finger | 50 | 1.21 |
| 2017-2020 | Foot | 51 | 1.23 |
| 2017-2020 | Forearm | 29 | 0.7 |
| 2017-2020 | Hamstring | 150 | 3.62 |
| 2017-2020 | Hand | 75 | 1.81 |
| 2017-2020 | Head | 35 | 0.84 |
| 2017-2020 | Heel | 8 | 0.19 |
| 2017-2020 | Hernia | 1 | 0.02 |
| 2017-2020 | Hip | 55 | 1.33 |
| 2017-2020 | Intercostal | 4 | 0.1 |
| 2017-2020 | Jaw | 4 | 0.1 |
| 2017-2020 | Knee | 140 | 3.38 |
| 2017-2020 | Leg | 23 | 0.55 |
| 2017-2020 | Neck | 58 | 1.4 |
| 2017-2020 | Oblique | 42 | 1.01 |
| 2017-2020 | Quadricep | 35 | 0.84 |
| 2017-2020 | Rib | 14 | 0.34 |
| 2017-2020 | Shin | 7 | 0.17 |
| 2017-2020 | Shoulder | 98 | 2.36 |
| 2017-2020 | Thigh | 2 | 0.05 |
| 2017-2020 | Thumb | 46 | 1.11 |
| 2017-2020 | Toe | 17 | 0.41 |
| 2017-2020 | Tricep | 8 | 0.19 |
| 2017-2020 | Wrist | 78 | 1.88 |

Appendix 14. Basketball Five Year Injury Incidence per Body Part

| Season | Body Part Injured | Count | Five Year Proportional Injury Incidence per 100 Players |
| --- | --- | --- | --- |
| 2007-2011 | Abdominal | 11 | 0.49 |
| 2007-2011 | Abductor | 3 | 0.13 |
| 2007-2011 | Achilles | 31 | 1.37 |
| 2007-2011 | Adductor | 69 | 3.04 |
| 2007-2011 | Ankle | 291 | 12.83 |
| 2007-2011 | Arm | 3 | 0.13 |
| 2007-2011 | Back | 163 | 7.19 |
| 2007-2011 | Bicep | 1 | 0.04 |
| 2007-2011 | Calf | 42 | 1.85 |
| 2007-2011 | Chest | 7 | 0.31 |
| 2007-2011 | Concussion | 32 | 1.41 |
| 2007-2011 | Elbow | 27 | 1.19 |
| 2007-2011 | Eye | 10 | 0.44 |
| 2007-2011 | Face | 6 | 0.26 |
| 2007-2011 | Fibula | 3 | 0.13 |
| 2007-2011 | Finger | 29 | 1.28 |
| 2007-2011 | Foot | 95 | 4.19 |
| 2007-2011 | Forearm | 3 | 0.13 |
| 2007-2011 | Hamstring | 61 | 2.69 |
| 2007-2011 | Hand | 20 | 0.88 |
| 2007-2011 | Head | 22 | 0.97 |
| 2007-2011 | Heel | 9 | 0.4 |
| 2007-2011 | Hernia | 8 | 0.35 |
| 2007-2011 | Hip | 53 | 2.34 |
| 2007-2011 | Jaw | 6 | 0.26 |
| 2007-2011 | Knee | 286 | 12.61 |
| 2007-2011 | Leg | 12 | 0.53 |
| 2007-2011 | Neck | 8 | 0.35 |
| 2007-2011 | Oblique | 3 | 0.13 |
| 2007-2011 | Patella | 19 | 0.84 |
| 2007-2011 | Quadricep | 31 | 1.37 |
| 2007-2011 | Rib | 11 | 0.49 |
| 2007-2011 | Shin | 6 | 0.26 |
| 2007-2011 | Shoulder | 70 | 3.09 |
| 2007-2011 | Thigh | 19 | 0.84 |
| 2007-2011 | Thumb | 35 | 1.54 |
| 2007-2011 | Toe | 30 | 1.32 |
| 2007-2011 | Wrist | 48 | 2.12 |
| 2012-2016 | Abdominal | 13 | 0.54 |
| 2012-2016 | Abductor | 5 | 0.21 |
| 2012-2016 | Achilles | 42 | 1.75 |
| 2012-2016 | Adductor | 61 | 2.54 |
| 2012-2016 | Ankle | 388 | 16.13 |
| 2012-2016 | Arm | 3 | 0.12 |
| 2012-2016 | Back | 181 | 7.53 |
| 2012-2016 | Bicep | 2 | 0.08 |
| 2012-2016 | Calf | 77 | 3.2 |
| 2012-2016 | Chest | 7 | 0.29 |
| 2012-2016 | Collarbone | 1 | 0.04 |
| 2012-2016 | Concussion | 46 | 1.91 |
| 2012-2016 | Elbow | 30 | 1.25 |
| 2012-2016 | Eye | 16 | 0.67 |
| 2012-2016 | Face | 2 | 0.08 |
| 2012-2016 | Fibula | 6 | 0.25 |
| 2012-2016 | Finger | 25 | 1.04 |
| 2012-2016 | Foot | 113 | 4.7 |
| 2012-2016 | Forearm | 3 | 0.12 |
| 2012-2016 | Hamstring | 92 | 3.83 |
| 2012-2016 | Hand | 42 | 1.75 |
| 2012-2016 | Head | 16 | 0.67 |
| 2012-2016 | Heel | 16 | 0.67 |
| 2012-2016 | Hernia | 5 | 0.21 |
| 2012-2016 | Hip | 87 | 3.62 |
| 2012-2016 | Jaw | 1 | 0.04 |
| 2012-2016 | Knee | 375 | 15.59 |
| 2012-2016 | Leg | 30 | 1.25 |
| 2012-2016 | Neck | 18 | 0.75 |
| 2012-2016 | Oblique | 3 | 0.12 |
| 2012-2016 | Patella | 11 | 0.46 |
| 2012-2016 | Quadricep | 37 | 1.54 |
| 2012-2016 | Rib | 21 | 0.87 |
| 2012-2016 | Shin | 9 | 0.37 |
| 2012-2016 | Shoulder | 107 | 4.45 |
| 2012-2016 | Thigh | 19 | 0.79 |
| 2012-2016 | Thumb | 30 | 1.25 |
| 2012-2016 | Toe | 40 | 1.66 |
| 2012-2016 | Tricep | 4 | 0.17 |
| 2012-2016 | Wrist | 49 | 2.04 |
| 2017-2020 | Abdominal | 5 | 0.32 |
| 2017-2020 | Achilles | 19 | 1.21 |
| 2017-2020 | Adductor | 32 | 2.04 |
| 2017-2020 | Ankle | 184 | 11.73 |
| 2017-2020 | Arm | 1 | 0.06 |
| 2017-2020 | Back | 76 | 4.85 |
| 2017-2020 | Calf | 22 | 1.4 |
| 2017-2020 | Collarbone | 1 | 0.06 |
| 2017-2020 | Concussion | 28 | 1.79 |
| 2017-2020 | Elbow | 17 | 1.08 |
| 2017-2020 | Eye | 7 | 0.45 |
| 2017-2020 | Face | 1 | 0.06 |
| 2017-2020 | Fibula | 3 | 0.19 |
| 2017-2020 | Finger | 11 | 0.7 |
| 2017-2020 | Foot | 48 | 3.06 |
| 2017-2020 | Forearm | 1 | 0.06 |
| 2017-2020 | Gluteus | 1 | 0.06 |
| 2017-2020 | Hamstring | 46 | 2.93 |
| 2017-2020 | Hand | 20 | 1.28 |
| 2017-2020 | Head | 9 | 0.57 |
| 2017-2020 | Heel | 15 | 0.96 |
| 2017-2020 | Hernia | 1 | 0.06 |
| 2017-2020 | Hip | 40 | 2.55 |
| 2017-2020 | Jaw | 2 | 0.13 |
| 2017-2020 | Knee | 174 | 11.1 |
| 2017-2020 | Leg | 14 | 0.89 |
| 2017-2020 | Neck | 14 | 0.89 |
| 2017-2020 | Oblique | 2 | 0.13 |
| 2017-2020 | Patella | 5 | 0.32 |
| 2017-2020 | Quadricep | 18 | 1.15 |
| 2017-2020 | Rib | 6 | 0.38 |
| 2017-2020 | Shin | 3 | 0.19 |
| 2017-2020 | Shoulder | 42 | 2.68 |
| 2017-2020 | Thigh | 11 | 0.7 |
| 2017-2020 | Thumb | 18 | 1.15 |
| 2017-2020 | Toe | 25 | 1.59 |
| 2017-2020 | Tricep | 1 | 0.06 |
| 2017-2020 | Wrist | 21 | 1.34 |

Appendix 15. Football Five Year Injury Incidence per Body Part

| Season | Body Part Injured | Count | Five Year Proportional Injury Incidence per 100 Players |
| --- | --- | --- | --- |
| 2007-2011 | Abdominal | 3 | 0.04 |
| 2007-2011 | Achilles | 6 | 0.09 |
| 2007-2011 | Adductor | 90 | 1.34 |
| 2007-2011 | Ankle | 238 | 3.56 |
| 2007-2011 | Arm | 4 | 0.06 |
| 2007-2011 | Back | 58 | 0.87 |
| 2007-2011 | Bicep | 6 | 0.09 |
| 2007-2011 | Calf | 51 | 0.76 |
| 2007-2011 | Chest | 26 | 0.39 |
| 2007-2011 | Collarbone | 1 | 0.01 |
| 2007-2011 | Concussion | 107 | 1.6 |
| 2007-2011 | Elbow | 20 | 0.3 |
| 2007-2011 | Eye | 3 | 0.04 |
| 2007-2011 | Face | 1 | 0.01 |
| 2007-2011 | Fibula | 11 | 0.16 |
| 2007-2011 | Finger | 15 | 0.22 |
| 2007-2011 | Foot | 83 | 1.24 |
| 2007-2011 | Forearm | 16 | 0.24 |
| 2007-2011 | Gluteus | 1 | 0.01 |
| 2007-2011 | Hamstring | 197 | 2.94 |
| 2007-2011 | Hand | 28 | 0.42 |
| 2007-2011 | Head | 48 | 0.72 |
| 2007-2011 | Heel | 4 | 0.06 |
| 2007-2011 | Hernia | 3 | 0.04 |
| 2007-2011 | Hip | 23 | 0.34 |
| 2007-2011 | Jaw | 4 | 0.06 |
| 2007-2011 | Knee | 386 | 5.77 |
| 2007-2011 | Leg | 8 | 0.12 |
| 2007-2011 | Neck | 42 | 0.63 |
| 2007-2011 | Quadricep | 33 | 0.49 |
| 2007-2011 | Rib | 14 | 0.21 |
| 2007-2011 | Shin | 4 | 0.06 |
| 2007-2011 | Shoulder | 92 | 1.37 |
| 2007-2011 | Thigh | 27 | 0.4 |
| 2007-2011 | Thumb | 13 | 0.19 |
| 2007-2011 | Toe | 29 | 0.43 |
| 2007-2011 | Tricep | 6 | 0.09 |
| 2007-2011 | Wrist | 15 | 0.22 |
| 2012-2016 | Abdominal | 11 | 0.17 |
| 2012-2016 | Achilles | 10 | 0.15 |
| 2012-2016 | Adductor | 93 | 1.41 |
| 2012-2016 | Ankle | 341 | 5.18 |
| 2012-2016 | Arm | 1 | 0.02 |
| 2012-2016 | Back | 62 | 0.94 |
| 2012-2016 | Bicep | 6 | 0.09 |
| 2012-2016 | Calf | 72 | 1.09 |
| 2012-2016 | Chest | 22 | 0.33 |
| 2012-2016 | Collarbone | 6 | 0.09 |
| 2012-2016 | Concussion | 295 | 4.48 |
| 2012-2016 | Elbow | 22 | 0.33 |
| 2012-2016 | Eye | 1 | 0.02 |
| 2012-2016 | Fibula | 6 | 0.09 |
| 2012-2016 | Finger | 8 | 0.12 |
| 2012-2016 | Foot | 124 | 1.88 |
| 2012-2016 | Forearm | 10 | 0.15 |
| 2012-2016 | Hamstring | 275 | 4.18 |
| 2012-2016 | Hand | 35 | 0.53 |
| 2012-2016 | Head | 16 | 0.24 |
| 2012-2016 | Hernia | 1 | 0.02 |
| 2012-2016 | Hip | 31 | 0.47 |
| 2012-2016 | Knee | 402 | 6.11 |
| 2012-2016 | Leg | 1 | 0.02 |
| 2012-2016 | Neck | 36 | 0.55 |
| 2012-2016 | Oblique | 1 | 0.02 |
| 2012-2016 | Quadricep | 36 | 0.55 |
| 2012-2016 | Rib | 32 | 0.49 |
| 2012-2016 | Shin | 4 | 0.06 |
| 2012-2016 | Shoulder | 127 | 1.93 |
| 2012-2016 | Thigh | 28 | 0.43 |
| 2012-2016 | Thumb | 16 | 0.24 |
| 2012-2016 | Toe | 38 | 0.58 |
| 2012-2016 | Tricep | 3 | 0.05 |
| 2012-2016 | Wrist | 8 | 0.12 |
| 2017-2020 | Abdominal | 9 | 0.24 |
| 2017-2020 | Achilles | 11 | 0.29 |
| 2017-2020 | Adductor | 54 | 1.43 |
| 2017-2020 | Ankle | 177 | 4.69 |
| 2017-2020 | Back | 39 | 1.03 |
| 2017-2020 | Bicep | 2 | 0.05 |
| 2017-2020 | Calf | 45 | 1.19 |
| 2017-2020 | Chest | 18 | 0.48 |
| 2017-2020 | Collarbone | 2 | 0.05 |
| 2017-2020 | Concussion | 182 | 4.82 |
| 2017-2020 | Elbow | 18 | 0.48 |
| 2017-2020 | Eye | 2 | 0.05 |
| 2017-2020 | Fibula | 5 | 0.13 |
| 2017-2020 | Finger | 1 | 0.03 |
| 2017-2020 | Foot | 64 | 1.69 |
| 2017-2020 | Forearm | 5 | 0.13 |
| 2017-2020 | Hamstring | 157 | 4.16 |
| 2017-2020 | Hand | 15 | 0.4 |
| 2017-2020 | Heel | 3 | 0.08 |
| 2017-2020 | Hip | 18 | 0.48 |
| 2017-2020 | Jaw | 1 | 0.03 |
| 2017-2020 | Knee | 227 | 6.01 |
| 2017-2020 | Neck | 35 | 0.93 |
| 2017-2020 | Oblique | 3 | 0.08 |
| 2017-2020 | Quadricep | 25 | 0.66 |
| 2017-2020 | Rib | 18 | 0.48 |
| 2017-2020 | Shin | 3 | 0.08 |
| 2017-2020 | Shoulder | 79 | 2.09 |
| 2017-2020 | Thigh | 9 | 0.24 |
| 2017-2020 | Thumb | 9 | 0.24 |
| 2017-2020 | Toe | 19 | 0.5 |
| 2017-2020 | Tricep | 3 | 0.08 |
| 2017-2020 | Wrist | 5 | 0.13 |

Appendix 16. Hockey Five Year Injury Incidence per Body Part

| Season | Body Part Injured | Count | Five Year Proportional Injury Incidence per 100 Players |
| --- | --- | --- | --- |
| 2007-2011 | Abdominal | 20 | 0.45 |
| 2007-2011 | Achilles | 2 | 0.05 |
| 2007-2011 | Adductor | 262 | 5.95 |
| 2007-2011 | Ankle | 126 | 2.86 |
| 2007-2011 | Arm | 32 | 0.73 |
| 2007-2011 | Back | 155 | 3.52 |
| 2007-2011 | Bicep | 1 | 0.02 |
| 2007-2011 | Calf | 4 | 0.09 |
| 2007-2011 | Chest | 9 | 0.2 |
| 2007-2011 | Collarbone | 3 | 0.07 |
| 2007-2011 | Concussion | 159 | 3.61 |
| 2007-2011 | Elbow | 11 | 0.25 |
| 2007-2011 | Eye | 30 | 0.68 |
| 2007-2011 | Face | 51 | 1.16 |
| 2007-2011 | Fibula | 1 | 0.02 |
| 2007-2011 | Finger | 49 | 1.11 |
| 2007-2011 | Foot | 145 | 3.29 |
| 2007-2011 | Forearm | 6 | 0.14 |
| 2007-2011 | Gluteus | 1 | 0.02 |
| 2007-2011 | Hamstring | 18 | 0.41 |
| 2007-2011 | Hand | 96 | 2.18 |
| 2007-2011 | Head | 149 | 3.39 |
| 2007-2011 | Heel | 1 | 0.02 |
| 2007-2011 | Hernia | 8 | 0.18 |
| 2007-2011 | Hip | 82 | 1.86 |
| 2007-2011 | Jaw | 16 | 0.36 |
| 2007-2011 | Knee | 277 | 6.29 |
| 2007-2011 | Leg | 142 | 3.23 |
| 2007-2011 | Neck | 46 | 1.05 |
| 2007-2011 | Oblique | 7 | 0.16 |
| 2007-2011 | Patella | 1 | 0.02 |
| 2007-2011 | Quadricep | 6 | 0.14 |
| 2007-2011 | Rib | 31 | 0.7 |
| 2007-2011 | Shin | 2 | 0.05 |
| 2007-2011 | Shoulder | 191 | 4.34 |
| 2007-2011 | Thigh | 13 | 0.3 |
| 2007-2011 | Thumb | 11 | 0.25 |
| 2007-2011 | Toe | 6 | 0.14 |
| 2007-2011 | Wrist | 56 | 1.27 |
| 2012-2016 | Abdominal | 5 | 0.11 |
| 2012-2016 | Achilles | 1 | 0.02 |
| 2012-2016 | Adductor | 71 | 1.62 |
| 2012-2016 | Ankle | 56 | 1.27 |
| 2012-2016 | Arm | 12 | 0.27 |
| 2012-2016 | Back | 53 | 1.21 |
| 2012-2016 | Bicep | 1 | 0.02 |
| 2012-2016 | Calf | 1 | 0.02 |
| 2012-2016 | Chest | 4 | 0.09 |
| 2012-2016 | Collarbone | 2 | 0.05 |
| 2012-2016 | Concussion | 128 | 2.91 |
| 2012-2016 | Elbow | 9 | 0.2 |
| 2012-2016 | Eye | 15 | 0.34 |
| 2012-2016 | Face | 31 | 0.71 |
| 2012-2016 | Fibula | 5 | 0.11 |
| 2012-2016 | Finger | 20 | 0.46 |
| 2012-2016 | Foot | 72 | 1.64 |
| 2012-2016 | Hamstring | 8 | 0.18 |
| 2012-2016 | Hand | 67 | 1.53 |
| 2012-2016 | Head | 63 | 1.43 |
| 2012-2016 | Heel | 2 | 0.05 |
| 2012-2016 | Hernia | 8 | 0.18 |
| 2012-2016 | Hip | 29 | 0.66 |
| 2012-2016 | Jaw | 11 | 0.25 |
| 2012-2016 | Knee | 113 | 2.57 |
| 2012-2016 | Leg | 61 | 1.39 |
| 2012-2016 | Neck | 30 | 0.68 |
| 2012-2016 | Oblique | 10 | 0.23 |
| 2012-2016 | Rib | 11 | 0.25 |
| 2012-2016 | Shoulder | 64 | 1.46 |
| 2012-2016 | Thigh | 3 | 0.07 |
| 2012-2016 | Thumb | 6 | 0.14 |
| 2012-2016 | Tricep | 1 | 0.02 |
| 2012-2016 | Wrist | 29 | 0.66 |
| 2017-2020 | Abdominal | 3 | 0.11 |
| 2017-2020 | Adductor | 23 | 0.87 |
| 2017-2020 | Ankle | 22 | 0.83 |
| 2017-2020 | Arm | 7 | 0.27 |
| 2017-2020 | Back | 25 | 0.95 |
| 2017-2020 | Bicep | 1 | 0.04 |
| 2017-2020 | Concussion | 75 | 2.84 |
| 2017-2020 | Elbow | 2 | 0.08 |
| 2017-2020 | Eye | 6 | 0.23 |
| 2017-2020 | Face | 12 | 0.45 |
| 2017-2020 | Fibula | 1 | 0.04 |
| 2017-2020 | Finger | 7 | 0.27 |
| 2017-2020 | Foot | 27 | 1.02 |
| 2017-2020 | Forearm | 1 | 0.04 |
| 2017-2020 | Hamstring | 3 | 0.11 |
| 2017-2020 | Hand | 32 | 1.21 |
| 2017-2020 | Head | 16 | 0.61 |
| 2017-2020 | Hip | 11 | 0.42 |
| 2017-2020 | Jaw | 5 | 0.19 |
| 2017-2020 | Knee | 39 | 1.48 |
| 2017-2020 | Leg | 28 | 1.06 |
| 2017-2020 | Neck | 13 | 0.49 |
| 2017-2020 | Oblique | 2 | 0.08 |
| 2017-2020 | Quadricep | 1 | 0.04 |
| 2017-2020 | Rib | 3 | 0.11 |
| 2017-2020 | Shoulder | 29 | 1.1 |
| 2017-2020 | Thumb | 4 | 0.15 |
| 2017-2020 | Wrist | 7 | 0.27 |

Appendix 17. Mean Difference by Sport per Body Part

| Injured Body Part | Baseball | Basketball | Football | Hockey |
| --- | --- | --- | --- | --- |
| Abdominal | 0.20205128 | 0.35266667 | 0.12333333 | 0.30622222 |
| Abductor | 0 | 0.21166667 | 0 | 0 |
| Achilles | 0.17484849 | 0.70307692 | 0.13333333 | 0 |
| Adductor | 0.52333333 | 0.9174359 | 0.52871795 | 2.82153846 |
| Ankle | 0.37538462 | 3.40615385 | 1.07333333 | 1.29230769 |
| Arm | 0.1780303 | 0.07533333 | 0.22 | 0.40181818 |
| Back | 1.0825641 | 2.09205128 | 0.37794872 | 1.58974359 |
| Bicep | 0.17076923 | 0.00666667 | 0.03309091 | 0.00666667 |
| Calf | 0.29051282 | 1.07179487 | 0.44153846 | 0.16 |
| Chest | 0.10836364 | 0.17111111 | 0.17102564 | 0.17333333 |
| Collarbone | 0.004 | 0.02 | 0.03714286 | 0.055 |
| Concussion | 0.16 | 0.96948718 | 2.32974359 | 1.37205128 |
| Elbow | 1.05923077 | 0.4574359 | 0.19846154 | 0.11527273 |
| Eye | 0.18410256 | 0.4 | 0.04666667 | 0.28742424 |
| Face | 0.13418182 | 0.1152381 | 0 | 0.53025641 |
| Fibula | 0.00666667 | 0.12444444 | 0.06945455 | 0.03933333 |
| Finger | 0.39641026 | 0.54589744 | 0.11133333 | 0.52128205 |
| Foot | 0.40564103 | 1.31512821 | 0.49769231 | 1.33717949 |
| Forearm | 0.23615385 | 0.08733333 | 0.11076923 | 0.094 |
| Gluteus | 0.04 | 0 | 0 | 0 |
| Hamstring | 0.63 | 0.86153846 | 1.12128205 | 0.22618182 |
| Hand | 0.53769231 | 0.61717949 | 0.24769231 | 0.70717949 |
| Head | 0.33641026 | 0.43846154 | 0.51388889 | 1.58538462 |
| Heel | 0.15136364 | 0.38128205 | 0.08666667 | 0.00666667 |
| Hernia | 0.14952381 | 0.12822222 | 0.15 | 0.14611111 |
| Hip | 0.33076923 | 1.20512821 | 0.23538462 | 0.92939394 |
| Intercostal | 0.03933333 | 0 | 0 | 0.17348485 |
| Jaw | 0.07933333 | 0.336 | 0.09333333 | 0 |
| Knee | 0.57820513 | 3.48897436 | 0.82 | 3.01794872 |
| Leg | 0.20128205 | 0.61015152 | 0.3 | 1.35974359 |
| Neck | 0.55974359 | 0.4619697 | 0.31102564 | 0.6269697 |
| Oblique | 0.51435897 | 0.13066667 | 0.07333333 | 0.155 |
| Patella | 0 | 0.68644444 | 0.23846154 | 0 |
| Quadricep | 0.41230769 | 0.72878788 | 0 | 0.07 |
| Rib | 0.28717949 | 0.45636364 | 0.24820513 | 0.37709091 |
| Shin | 0.22282051 | 0.14644444 | 0.03277778 | 0.01 |
| Shoulder | 1.01102564 | 1.38230769 | 0.86358974 | 2.04128205 |
| Thigh | 0.08327273 | 0.41128205 | 0.28025641 | 0.15428571 |
| Thumb | 0.40871795 | 0.55128205 | 0.11742424 | 0.11166667 |
| Toe | 0.25128205 | 0.77666667 | 0.16871795 | 0.12 |
| Tricep | 0.13923077 | 0.3 | 0.03944444 | 0 |
| Wrist | 0.49153846 | 0.82230769 | 0.11530303 | 0.64948718 |

Appendix 18. Five Year Mean Difference by Sport per Body Part

| Injured Body part | Baseball | Basketball | Football | Hockey |
| --- | --- | --- | --- | --- |
| Abdominal | 0.03333333 | 0.14666667 | 0.13333333 | 0.22666667 |
| Abductor | 0.01 | 0.08 | 0 | 0 |
| Achilles | 0.1 | 0.36 | 0.13333333 | 0.03 |
| Adductor | 0.34 | 0.66666667 | 0.06 | 3.38666667 |
| Ankle | 0.35333333 | 2.93333333 | 1.08 | 1.35333333 |
| Arm | 0.2 | 0.04666667 | 0.04 | 0.30666667 |
| Back | 0.94 | 1.78666667 | 0.10666667 | 1.71333333 |
| Bicep | 0.09333333 | 0.04 | 0.02666667 | 0.01333333 |
| Calf | 0.17333333 | 1.2 | 0.28666667 | 0.07 |
| Chest | 0.05333333 | 0.02 | 0.1 | 0.11 |
| Collarbone | 0.00666667 | 0.02 | 0.05333333 | 0.02 |
| Concussion | 0.04666667 | 0.33333333 | 2.14666667 | 0.51333333 |
| Elbow | 1.12 | 0.11333333 | 0.12 | 0.11333333 |
| Eye | 0.10666667 | 0.15333333 | 0.02 | 0.3 |
| Face | 0.05333333 | 0.13333333 | 0 | 0.47333333 |
| Fibula | 0.03 | 0.08 | 0.04666667 | 0.06 |
| Finger | 0.14 | 0.38666667 | 0.12666667 | 0.56 |
| Foot | 0.41333333 | 1.09333333 | 0.42666667 | 1.51333333 |
| Forearm | 0.16666667 | 0.04666667 | 0.07333333 | 0.1 |
| Gluteus | 0 | 0 | 0 | 0 |
| Hamstring | 0.79333333 | 0.76 | 0.82666667 | 0.2 |
| Hand | 0.66666667 | 0.58 | 0.08666667 | 0.64666667 |
| Head | 0.26 | 0.26666667 | 0.48 | 1.85333333 |
| Heel | 0.04666667 | 0.37333333 | 0.02 | 0.03 |
| Hernia | 0.06666667 | 0.19333333 | 0.02 | 0 |
| Hip | 0.35333333 | 0.85333333 | 0.09333333 | 0.96 |
| Intercostal | 0.06 | 0 | 0.03 | 0.11333333 |
| Jaw | 0.05333333 | 0.14666667 | 0 |  |
| Knee | 0.45333333 | 2.99333333 | 0.22666667 | 3.20666667 |
| Leg | 0.18 | 0.48 | 0.1 | 1.44666667 |
| Neck | 0.40666667 | 0.36 | 0.25333333 | 0.37333333 |
| Oblique | 0.28 | 0.00666667 | 0.06 | 0.1 |
| Patella | 0 | 0.34666667 | 0 | 0 |
| Quadricep | 0.17333333 | 0.26 | 0.11333333 | 0.1 |
| Rib | 0.22 | 0.32666667 | 0.18666667 | 0.39333333 |
| Shin | 0.16 | 0.12 | 0.01333333 | 0 |
| Shoulder | 0.82 | 1.18 | 0.48 | 2.16 |
| Thigh | 0.04 | 0.09333333 | 0.12666667 | 0.23 |
| Thumb | 0.26 | 0.26 | 0.03333333 | 0.07333333 |
| Toe | 0.04 | 0.22666667 | 0.1 | 0 |
| Tricep | 0.07333333 | 0.11 | 0.02666667 | 0 |
| Wrist | 0.55333333 | 0.52 | 0.06666667 | 0.66666667 |
